# C-3 Steroidal Hemiesters as Inhibitors of 17β-Hydroxysteroid Dehydrogenase Type 10

**DOI:** 10.1021/acsomega.3c10148

**Published:** 2024-02-28

**Authors:** Michaela Hanzlova, Barbora Slavikova, Marina Morozovova, Kamil Musilek, Aneta Rotterova, Lucie Zemanová, Eva Kudova

**Affiliations:** † Faculty of Science, Department of Chemistry, University of Hradec Kralove, Rokitanskeho 62, 500 03 Hradec Kralove, Czech Republic; ‡ Institute of Organic Chemistry and Biochemistry, Czech Academy of Sciences, Flemingovo namesti 2, Prague 6 166 10, Czech Republic

## Abstract

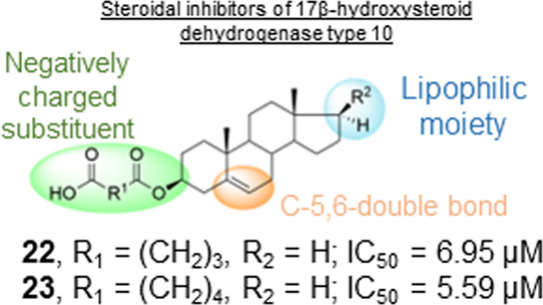

17β-HSD10 is a mitochondrial enzyme that catalyzes the steroidal oxidation of a hydroxy group to a keto group and, thus, is involved in maintaining steroid homeostasis. The druggability of 17β-HSD10 is related to potential treatment for neurodegenerative diseases, for example, Alzheimer’s disease or cancer. Herein, steroidal derivatives with an acidic hemiester substituent at position C-3 on the skeleton were designed, synthesized, and evaluated by using pure recombinant 17β-HSD10 converting 17β-estradiol to estrone. Compounds **22** (IC_50_ = 6.95 ± 0.35 μM) and **23** (IC_50_ = 5.59 ± 0.25 μM) were identified as the most potent inhibitors from the series. Compound **23** inhibited 17β-HSD10 activity regardless of the substrate. It was found not cytotoxic toward the HEK-293 cell line and able to inhibit 17β-HSD10 activity also in the cellular environment. Together, these findings support steroidal compounds as promising candidates for further development as 17β-HSD10 inhibitors.

## Introduction

Human 17β-hydroxysteroid dehydrogenase type 10 (17β-HSD10, SDR5C1 also called ABAD/ERAB or HADH2, UniProt ID Q99714) is a member of the short-chain dehydrogenase/reductase (SDR) superfamily that is expressed in the mitochondrial matrix in a variety of tissues, such as lung, liver, brain, and other.^1^ The 17β-HSD10 is a so-called moonlighting enzyme that exhibits at least two physiologically relevant functions. First, it is a key component of the ribonuclease P (RNase P) complex that participates in isoleucine metabolism as well as in lipid metabolism.^2^ The second essential function reflecting its name is its role in steroid metabolism. 17β-HSD10 catalyzes, e.g., NAD^+^-dependent oxidation of 17β-estradiol to less active estrone, 3α-androstanediol to more potent 5α-dihydrotestosterone, or neurosteroid allopregnanolone to 5α-dihydroprogesterone. Thus, the activity of 17β-HSD10 can influence important cellular processes related to the steroid concentrations, such as proliferation, apoptosis, or neural excitability.^3,4^ Finally, the crucial role of 17β-HSD10 in the development of various pathological conditions and diseases should be mentioned. According to the literature, the 17β-HSD10 overexpression may lead to the disturbance in steroid homeostasis proposed as an important factor attributing to the development of various diseases, such as Alzheimer′s disease,^5^ prostate cancer,^6,7^ or osteosarcoma.^8^ Taken together, the restoration of steroid homeostasis through 17β-HSD10 inhibition is thought to promote neuroprotection and serve as a promising therapeutic approach for the development of novel drug-like molecules.^5,8,9^

To date, several groups of 17β-HSD10 inhibitors have been described in the literature. For details, see the review of Vinklarova et al.^10^ They can be divided into several groups based on their structure, namely, benzothiazole-based ureas,^11−15^ pyrazole-pyrimidine compounds,^16,17^ steroidal inhibitors,^18−20^ and risperidone or its analogs.^21^ Based on the mechanism of action, inhibitors of 17β-HSD10 are designed to either block the catalytic activity of the enzyme or modulate the interaction of 17β-HSD10 with amyloid-β (Aβ) that is contributing to Aβ-induced toxicity by promoting mitochondrial dysfunction.

**Scheme 1:**
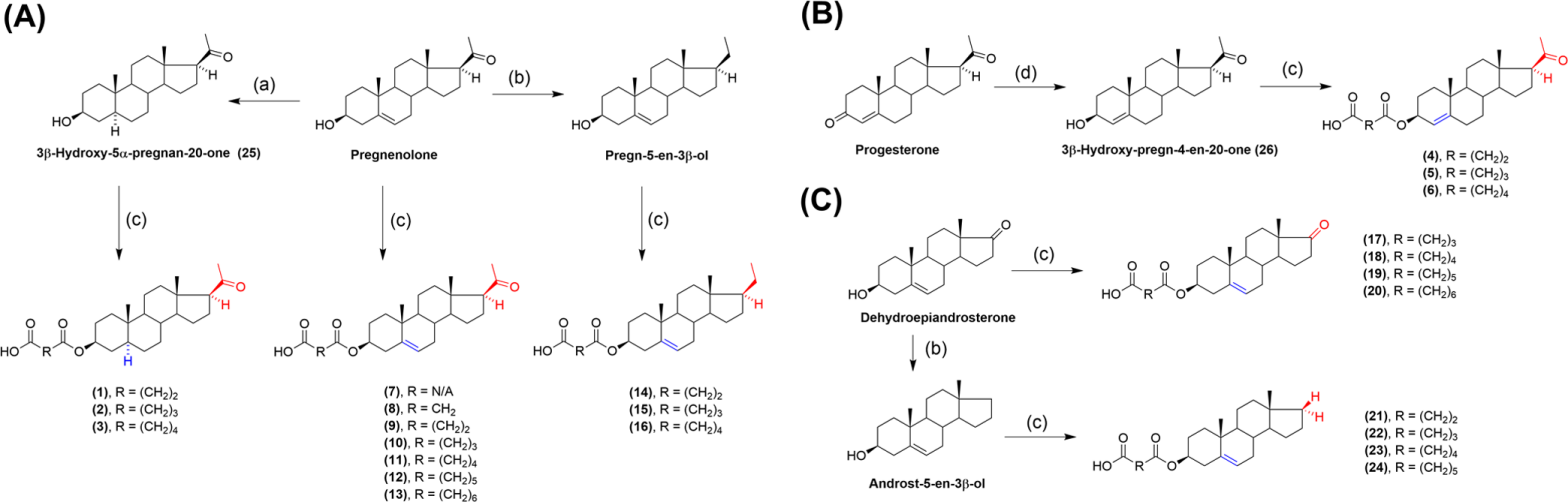
Synthesis of Compounds **1**–**24** Reaction conditions and reagents: (a) H_2_, Pd/C, EtOH/EtOAc; (b) Zn, TMSCl, MeOH, DCM; (c) Compound **7**: oxalyl chloride, Et_3_N, DCM, DMF. Compound **8**: 2,2-dimethyl-1,3-dioxane-4,6-dione, toluene, 80 °C. Compounds **1**, **4**, **9**, **14**, and **21**: succinic anhydride, 4-dimethylaminopyridine, pyridine, 110 °C. Compounds **2**, **5**, **10**, **15**, **17**, and **22**: glutaric anhydride, 4-dimethylaminopyridine, pyridine, 110 °C. Compounds **3**, **6**, **11**, **16**, **18**, and **23**: adipic acid, 1-ethyl-3-(3-dimethylaminopropyl)carbodiimide, N,N-diisopropylethylamine, 4-dimethylaminopyridine, DCM. Compounds **12**, **19**, and **24**: heptanedioic acid, 1-ethyl-3-(3-dimethylaminopropyl)carbodiimide, N,N-diisopropylethylamine, 4-dimethylaminopyridine, DCM. Compounds 13 and 20: suberic acid, 1-ethyl-3-(3-dimethylaminopropyl)carbodiimide, N,N-diisopropylethylamine, 4-dimethylaminopyridine, DCM; (d) NaBH_4_, CeCl_3_·7H_2_O, MeOH.

According to the literature, pyrazole-pyrimidine compound AG18051 is a potent inhibitor of 17β-HSD10.^16^ Co-crystallization of AG18051 with human 17β-HSD10 with its NAD^+^ cofactor has shown that it forms a covalent adduct with amino acids in the active site. Nevertheless, AG18051 has been further studied,^22^ and attempts for rational optimization studies of the structure have been also published.^17^ Very recently, a novel benzothiazolylurea inhibitor with similar efficiency and noncompetitive mode of action has been described.^15^ The most potent steroidal structures that have been described so far are RM-532-46 and D-3,7.^18,20^ Interestingly, compounds RM-532–46, D-3,7, as well as benzothiazole-based urea inhibitors are nitrogen-containing compounds that potentially can act as a proton acceptor (a base).^11−13^ Therefore, we proposed, synthesized, and tested a series of negatively charged steroids **1**–**24** (Scheme 1) for their ability to inhibit 17β-HSD10 activity.

Synthesis of neurosteroids for the treatment of yet untreatable central nervous system diseases represents a novel and attractive target for the development of drug-like compounds due to their endogenous origin and significantly more inherent nature to humans than, e.g., benzothiazole-based ureas or pyrazole-pyrimidine compounds. In addition, recently FDA-approved neurosteroids brexanolone (treatment of postpartum depression)^23,24^ and ganaxolone (treatment of seizures in CDKL5 deficiency)^25^ provide evidence of still underappreciated possibilities of neurosteroids in drug development.

## Results and Discussion

### Chemistry

The synthesis of compounds **1**–**24** is shown in Scheme 1. Compounds **1**–**3** were prepared from commercially available pregnenolone (Steraloids, Newport, RI). Compound 3β-hydroxy-5α-pregnan-20-one **25** was prepared by catalytic hydrogenation with Pd/C of pregnenolone.^26,27^ Compounds **4**–**6** were prepared from commercially available progesterone (Steraloids, Newport, RI). The conjugated carbonyl group of progesterone in position C-3 was selectively reduced by using sodium borohydride in the presence of cerium(III) chloride (Luche reduction) affording compound **26** in 50% yield.^28^ Compounds **1**–**24** were prepared by treatment of the parent C-3 hydroxyl group with anhydride or carboxylic acid depending on the availability of such reagents. In brief, compounds **1** and **4** were esterified with succinic anhydride in the presence of DMAP in pyridine at 110 °C, affording compounds **25** and **26** in 65 and 40% yield, respectively. Compounds **2** and **5** were prepared from compounds **25** and **26** by treatment with glutaric anhydride in the presence of DMAP in pyridine at 110 °C. Hemiesters **2** (45% yield) and **5** (62% yield) were obtained. The treatment of compounds **25** and **26** with adipic acid, 1-ethyl-3-(3-(dimethylamino)propyl)carbodiimide (EDCI), *N,N*-diisopropylethylamine (DIPEA), and DMAP in DCM gave hemiesters **3** and **6** (93% and 51% yield). Compounds **7**–**24** were prepared analogously as compounds **1**–**6** according to the literature^29^ from pregnenolone, dehydroepiandrosterone, pregn-5-en-3β-ol, and androst-5-en-3β-ol. Decarbonylation was achieved by Clemmensen reduction mediated by Zn/TMSCl according to the literature.^30^

### Biological Results

The inhibitory effect of compounds **1**–**24** at 10 μM concentration was evaluated *in vitro* using the conversion of 17β-estradiol to estrone by purified recombinant 17β-HSD10. As standards, we used the known inhibitor AG18051 as the positive control and the published steroidal compounds,^18^ namely, pregnenolone, testosterone, dihydrotestosterone, androsterone, epiandrosterone, and dehydroepiandrosterone. Our study has shown that the uncharged steroids that were used as comparators were inactive or displayed a very low inhibitory efficiency in the assay (Table S1). These results reflect the results of the study of Ayan et al.^18^ although they used a different (cellular) assay.

**Figure 1:**
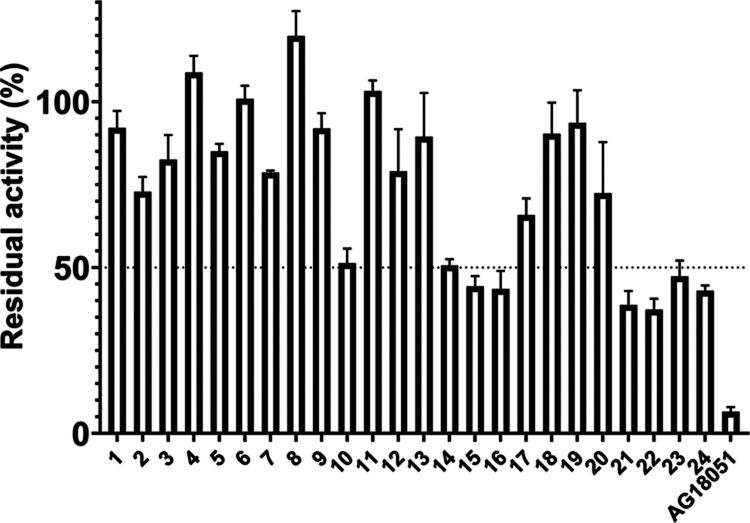
Evaluation of the inhibitory effect of compounds **1**–**24** and inhibitor AG18051 (standard) at 10 μM. Values represent means ± SD (*n* = 4).

Further, we tested three series of steroidal compounds of 20-oxo-pregna(e)ne skeleton that differed at position C-5 by presence/absence of a double bond: (i) compounds **1**–**3** with 5α-stereochemistry, (ii) unsaturated compounds **4**–**6** with C-4,5 double bond (Δ^4^ compounds), and (iii) unsaturated compounds **7**–**10** with C-5,6 double bond (Δ^5^ compounds). In the primary screening, only compound **10** demonstrated an inhibitory ability of approximately 50% at a 10 μM concentration (Figure 1). Thus, considering the structure of compound **10**, we have prepared Δ^5^ compounds varying with the substituent at position C-17 with a C-3 linker of various lengths: (i) Δ^5^-20-oxo compounds **11**–**13** with longer C-3 linker than compound **10**, (ii) Δ^5^-20-deoxy compounds **14**–**16** without 20-oxo substituent, (iii) Δ^5^-17-oxo compounds **17**–**20** belonging to androstane family having C-17 substituent, and (iv) Δ^5^ compounds **21**–**24** without substituent at position C-17.

Interestingly, the structure–activity relationship revealed that compounds with lipophilic substitution at position C-17, namely **14**–**16** and **21**–**24**, were the most active compounds in the study. In particular, steroids bearing 20-oxo (**1**–**13**) or 17-oxo substituent (**17**–**20**) demonstrated an inhibitory ability of ≤50% at 10 μM concentration except for compound **10** which demonstrated approximately 50% inhibition. In contrast, decarbonylation at position C-20 (**14**–**16**) and position C-17 (**21**–**24**) afforded an inhibitory effect of ≥50% at 10 μM concentration (Figure 1). Therefore, compounds **10**, **14**–**16**, and **21**–**24** were further described by their IC_50_ values. The results are summarized in Table 1. All tested compounds **10**, **14**–**16**, and **21**–**24** displayed inhibition with IC_50_ values varying from 5.59 μM (compound **23**) to 16.89 μM (compound **10**). Dose–response curves for the most potent inhibitors **22** (IC_50_ ∼6.95 μM) and **23** (IC_50_ ∼5.59 μM) are shown in Figure S25A,B. The most potent inhibitor **23** was also tested for its ability to inhibit 17β-HSD10 activity regardless of the substrate. In particular, the oxidation of steroid allopregnanolone was also inhibited by compound **23** with IC_50_ ∼15.25 μM. The dose–response curve is shown in Figure S25C.

**Table 1:** IC_50_ Values for Compounds **10**, **14**–**16**, and **21**–**24**^a^

compound	IC_50_ (μM)
**10**	16.89 ± 1.33
**14**	9.94 ± 0.23
**15**	9.01 ± 0.43
**16**	9.29 ± 0.28
**21**	7.33 ± 0.45
**22**	6.95 ± 0.35
**23**	5.59 ± 0.25
**24**	7.17 ± 0.40
**AG18051**	0.09 ± 0.01^15^

a Values represent means ± SEM (*n* = 4).

It should be noted that the IC_50_ values determined in our experiments using the purified recombinant 17β-HSD10 cannot be directly compared with other published studies due to the strong dependency of IC_50_ value on various factors of the assay, i.e., the amount of enzyme, particular substrate, or other experimental conditions. For example, steroid inhibitor RM-532-46 has been demonstrated as a strong inhibitor (IC_50_ = 0.55 μM) in the cellular assay using the HEK-293 cells.^18^ The study of Boutin et al.^19^ used a method to a certain extent comparable to our assay (*in vitro* conditions, recombinant enzyme, identical substrate) and described compound RM-532-46 with IC_50_ of 610 μM using 17β-estradiol and 235 μM using allopregnanolone as substrates. However, no standard (e.g., AG18051) was used in these studies. Structurally diverse inhibitors of 17β-HSD10 of the benzothiazolyl type have been described as compounds having IC_50_ values in the submicromolar to 1 μM concentration range. Only one compound (**26**) was able to achieve the potency of AG18051, namely, a derivative of benzothiazole-based urea with the IC_50_ value of 0.07 μM. Unfortunately, this compound was identified as toxic for the HEK-293 cell line.^15^

**Figure 2:**
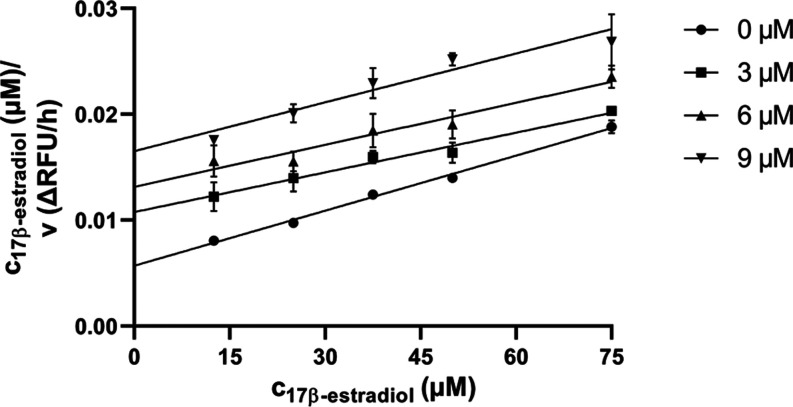
Kinetic inhibition of 17β-HSD10 using compound **23** at 0, 3, 6, and 9 μM concentrations. Data are shown as mean ± SD (*n* = 3).

The most potent inhibitor from the current series, compound **23** was further evaluated for the type of inhibition. The uninhibited enzymatic reaction (0 μM **23**) was compared with the enzymatic reaction at three concentrations of compound **23** (3, 6, and 9 μM) using 17β-estradiol as the substrate. We have hypothesized that a compound of steroid origin could demonstrate a competitive mechanism of action. Such inhibitor binds the enzyme preventing the formation of the enzyme–substrate complex. Thus, the substrate concentration does not enhance the inhibitory ability. This hypothesis was confirmed by the kinetic experiment. The obtained data were linearized by using Hanes–Woolf plot (Figure 2).

Considering the expected biological instability of hemiester group and lipophilicity of compounds **14**–**16** and **21**–**24**, the results of permeability and plasma stability experiments were included in this study (Table 2).^31^ According to the literature, drugs with permeability coefficients greater than 1 × 10^–6^ cm/s in Caco-2 cells are expected to be completely absorbed in humans.^32,33^ Therefore, we conclude that compounds **14**–**16** and **21**–**24** display sufficient permeability. Given the presence of the metabolically unstable hemiester moiety, compounds **14**–**16** and **21**–**24** were expected to exhibit poor stability in both rat and human plasma. The stability was described as the percentage of the parental compound remaining in plasma after 8 h. Surprisingly, all compounds **14**–**16** and **21**–**24**^31^ demonstrated high stability in human plasma after 8 h of incubation and mediocre to high stability in rat plasma (Table 2). We conclude that such unexpected stability in plasma offers new potential for the structure–activity relationship study targeting the stability of the hemiester moiety at position C-3.

**Table 2:** *In Vitro* Safety Assessment and Plasma Stability of Tested Compounds **14**–**16** and **21**–**24**

	Caco-2 cell permeability (50 μM)	rat plasma stability	human plasma stability
compound	Papp (cm/s)	% remaining after 8 h	
**14**	5.7 × 10^–6^	55	100
**15**	1.9 × 10^–6^	99	100
**16**	1.2 × 10^–6^	43	94
**21**	1.0 × 10^–4^	88	99
**22**	1.2 × 10^–5^	57	98
**23**	3.1 × 10^–6^	32	100
**24**	1.3 × 10^–6^	38	100

**Figure 3:**
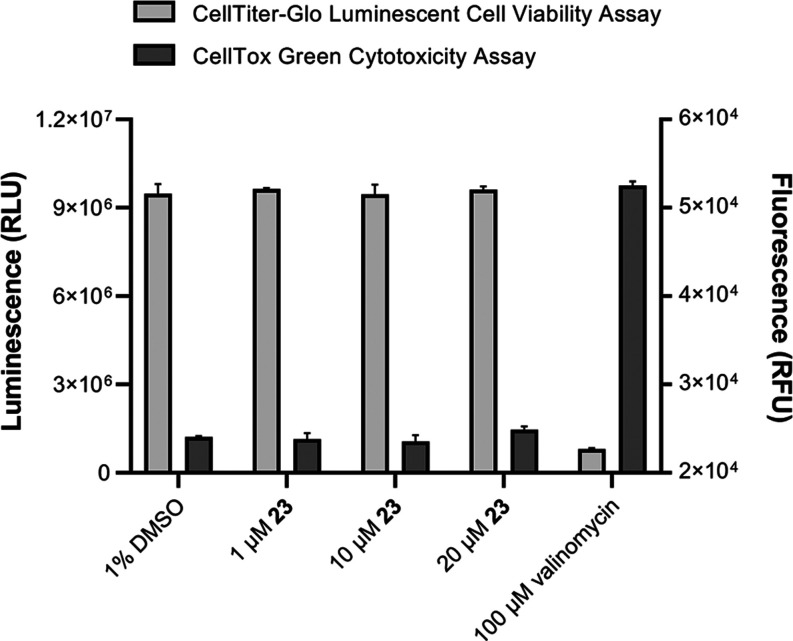
Viability of HEK-293 cells in the presence of compound **23** (measured by the CellTiter-Glo Viability Assay) and its cytotoxic effect (measured by the CellTox Green Assay). As controls for the cell viability, DMSO-treated cells were used. As a control for cytotoxicity, valinomycin-treated cells were used. Data are presented as mean ± SD (*n* = 3).

Finally, because the strongest inhibitor identified *in vitro* compound **23** has a favorable permeability coefficient, its potency to inhibit 17β-HSD10 also at the cellular level was determined. First, it was necessary to exclude the possibility that higher concentrations of compound **23** could be unfavorable to cells. The effects of compound **23** on the cellular viability and cytotoxicity of this compound (1, 10, and 20 μM) were determined using CellTiter-Glo Viability Assay and CellTox Green Assay, respectively, on HEK-293 cell line. The data in Figure 3 show that compound **23** does not influence HEK-293 viability and is not toxic to HEK-293 cell line up to a concentration of 20 μM.

**Figure 4:**
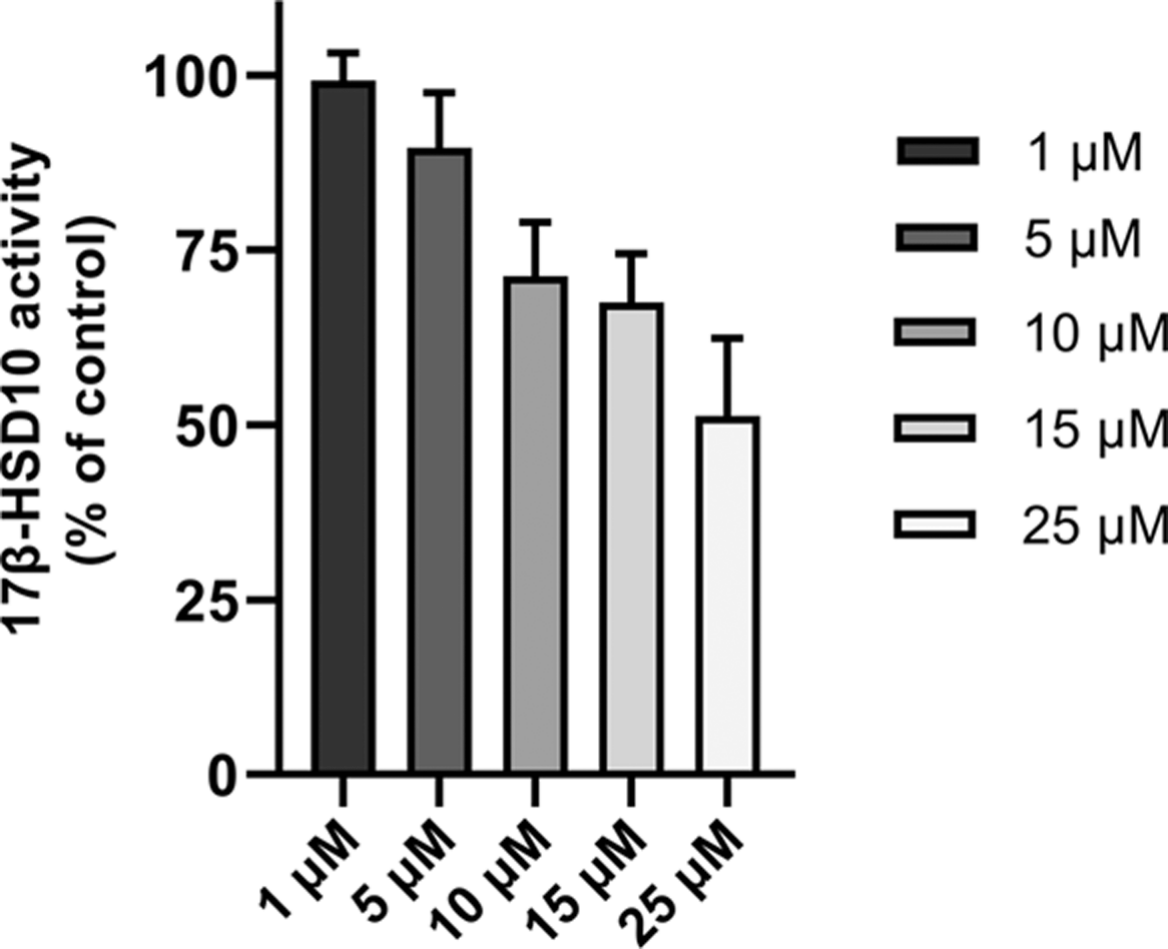
Inhibition of 17β-HSD10 by compound **23** in an HEK-293 cellular model overexpressing 17β-HSD10 (HEK-293-HSD10). The activity of 17β-HSD10 was determined using a probe (−)–CHANA as a substrate, and the fluorescence of formed product CHANK was measured. Values are given as mean ± SD from two biological replicates with three technical replicates.

Identical concentrations of compound **23** were used for fluorogenic cellular assay using a special probe (−)–CHANA as a substrate for 17β-HSD10 overexpressed in HEK-293 (HEK-293-HSD10 cell line).^15^ These results demonstrate that compound **23** can inhibit 17β-HSD10 in such a complex system. Its inhibition ability is dose-dependent, and the highest concentration used (25 μM) leads to ∼50% inhibition of the activity (Figure 4). The exact IC_50_ value at the cellular level was not possible to determine because of some method interferences, as well as its unknown cytotoxic characteristics. Higher IC_50_ (>20 μM) is in agreement with the high concentration of 17β-HSD10 in the model cell line.^15^

The results of our study have described the structure–activity relationship for steroids with the C-3 hemiester moiety to the inhibitory effect on 17β-HSD10 *in vitro*. We have shown the ability of the nontoxic compound **23** to inhibit the target enzyme also on the cellular level, along with cellular permeability and stability in rat and human plasma.

## Conclusions

Taken together, from the results of our study using both steroidal standards from Ayan et al.^18^ and the standard inhibitor AG18051, it can be concluded that steroidal compounds of the androstane skeleton (**14**–**16** and **22**–**24**) bearing a lipophilic substitution at the position C-17 with a negatively charged hemiester moiety at C-3 of various lengths (C_2_–C_7_) demonstrate promising *in vitro* inhibitory potential and they could serve as a useful tool for further development of steroidal 17β-HSD10 inhibitors.

## Materials and Methods

### Chemistry

All of the other commercial reagents and solvents were used without purification. Melting points were measured with a Micro Processor Melting-point apparatus (Hund/Wetzlar, Germany). Elemental analysis was measured with a PE 2400 Series II CHNS/O Analyzer (PerkinElmer, MA). Samples were prepared with a microbalance MX5 (Mettler Toledo, Switzerland). Optical rotation was measured with AUTOPOL IV (Rudolph Research Analytical, NJ) at 20 °C at 589 nm. ^1^H and ^13^C NMR spectra were measured in a Bruker AVANCE III 400 MHz. Coupling constants (*J*) are given in Hz. The HR-MS spectra were measured with an LTQ Orbitrap XL (Thermo Fischer Scientific, MA). Flash chromatography was performed with a puriFlash 5.250 instrument (Interchim, France) using neutral silica gel (Merck, 40–63 μm) and an ELSD detector.

#### General Procedure I: Synthesis of Steroidal Hemiesters from Anhydrides

A mixture of steroid (1 mmol) and dicarboxylic acid anhydride (6 equiv) was dried overnight at 50 °C. Then, dry pyridine (12 mL) and DMAP (0.24 equiv) were added. The mixture was stirred at 110 °C for 6 h under an inert atmosphere. The reaction mixture was then poured into water and extracted with DCM. Combined organic extracts were washed with brine and dried over sodium sulfate. Solvents were evaporated, and the residue was purified on a column of silica gel.

A mixture of predried steroid (1 mmol) and dicarboxylic acid anhydride (6 equiv), dry pyridine (12 mL), and DMAP (0.24 equiv) was stirred at 110 °C for 6 h under an inert atmosphere. The reaction mixture was extracted with DCM (3×). The combined organic extracts were washed with brine (2×) and dried over sodium sulfate. The evaporated crude product was purified on a silica gel column.

#### General Procedure II: Synthesis of Steroidal Hemiesters from ω-Dicarboxylic Acids

To a solution of dicarboxylic acid (2 mmol), DIPEA (2 mmol), EDCI (2 mmol), and DMAP (0.24 equiv) in dry DCM (10 mL) was added a solution of steroid (1 mmol) in dry DCM (5 mL) at 0 °C under an inert atmosphere. After 18 h, the solvents were evaporated. The residue was dissolved in DCM, washed with brine, and dried over sodium sulfate. The residue was purified on a column of silica gel.

##### 20-Oxo-5α-pregnan-3β-yl Hemisuccinate **1**

Compound **1** (270 mg, 65%) was prepared by *General Procedure I* from 3β-hydroxy-5α-pregnan-20-one **25** (318.5 mg, 1.0 mmol) by column chromatography (30–50% ethyl acetate in petroleum ether): mp 196–198 °C (toluene); [α]_D_^20^ +70.6 (*c* 0.2, CHCl_3_). ^1^H NMR (400 MHz, CDCl_3_): δ 0.60 (s, 3H, H-18), 0.82 (3H, s, H-19), 2.11 (3H, s, H-21), 2.52 (1H, t, *J* = 8.9, H-17), 2.63 (4H, m, H-2′ and H-3′), 4.72 (1H, tt, *J*_1_ = 11.3, *J*_2_ = 4.9, H-3). ^13^C NMR (101 MHz, CDCl_3_): δ 209.96, 177.46, 171.84, 74.34, 63.96, 56.76, 54.20, 44.77, 44.390, 39.15, 36.86, 35.64, 35.59, 34.00, 32.05, 31.68, 29.41, 29.05, 28.58, 27.49, 24.54, 22.94, 21.34, 13.60, 12.35. IR (CHCl_3_): 1727, 1716, 1702 (C=O); 1179 (C–O). MS ESI *m*/*z*: 417.2 (100%, M – 1), 418.3 (32%, M). HR-MS (ESI) *m*/*z* for C_25_H_37_O_5_ [M – 1] calcd 417.26465, found 417.26475. For C_25_H_38_O_5_ (418.6) calcd: 71.74%, C; 9.15% H. Found: 71.70%, C; 9.22%, H.

##### 20-Oxo-5α-pregnan-3β-yl Hemiglutarate **2**

Compound **2** (195 mg, 45%) was prepared by *General Procedure I* from 3β-hydroxy-5α-pregnan-20-one **25** (318.5 mg, 1.0 mmol) by column chromatography (30–50% ethyl acetate in petroleum ether): mp 165–167 °C (EtOAc); [α]_D_^20^ 63.9 (*c* 0.310, CHCl_3_). ^1^H NMR (400 MHz, CDCl_3_): δ 0.60 (3H, s, H-18), 0.82 (3H, s, H-19), 1.94 (2H, p, *J* = 7.3, H-3′), 2.11 (3H, s, H-21), 2.36 (2H, t, *J* = 7.3, H-2′), 2.42 (2H, p, *J* = 7.3, H-4′), 2.52 (1H, t, *J* = 9.0, H-17), 4.70 (1H, tt, *J*_1_ = 11.4, *J*_2_ = 4.9, H-3). ^13^C NMR (101 MHz, CDCl_3_): δ 209.96, 178.30, 172.58, 73.90, 63.96, 56.76, 54.21, 44.78, 44.39, 39.15, 36.88, 35.65, 35.59, 34.10, 33.72, 33.01, 32.05, 31.68, 28.59, 27.59, 24.54, 22.93, 21.34, 20.08, 13.60, 12.36. IR (CHCl_3_): 1722, 1711, 1703 (C=O); 1192 (C–O). MS ESI *m*/*z*: 431.3 (100%, M – 1), 432.3 (32%, M). HR-MS (ESI) *m*/*z* for C_26_H_39_O_5_ [M – 1] calcd 431.28030, found 431.27994. For C_26_H_40_O_5_ (432.6) calcd: 72.19%, C; 9.32% H. Found: 71.95%, C; 9.40%, H.

##### 20-Oxo-5α-pregnan-3β-yl Hemiadipate **3**

Compound **3** (416 mg, 93%) was prepared by *General Procedure II* from 3β-hydroxy-5α-pregnan-20-one **25** (318.5 mg, 1.0 mmol) by column chromatography (20–30% ethyl acetate in petroleum ether): mp 127–130 °C (EtOAc); [α]_D_^20^ 60.1 (*c* 0.284, CHCl_3_). ^1^H NMR (400 MHz, CDCl_3_): δ 0.60 (3H, s, H-18), 0.82 (3H, s, H-19), 2.11(3H, s, H-21), 2.25–2.41 (4H, m, H-2′ and H-5′), 2.52 (1H, t, *J* = 8.9, H-17), 4.70 (1H, tt, *J*_1_ = 11.4, *J*_2_ = 4.9, H-3). ^13^C NMR (101 MHz, CDCl_3_): δ 209.94, 178.66, 173.03, 73.73, 63.96, 56.76, 54.22, 44.78, 44.39, 39.16, 36.89, 35.65, 35.60, 34.42, 34.11, 33.63, 32.05, 31.68, 28.60, 27.59, 24.54, 24.21, 22.93, 21.34, 13.60, 12.36. IR (CHCl_3_): 1723, 1709, 1704 (C=O); 1191, 1182 (C–O). MS ESI *m*/*z*: 445.3 (100%, M – 1), 446.3 (30%, M). HR-MS (ESI) *m*/*z* for C_27_H_41_O_5_ [M – 1] calcd 445.29595, found 445.29586. For C_27_H_42_O_5_ (446.6) calcd: 72.61%, C; 9.48% H. Found: 72.82%, C; 9.61%, H.

##### 20-Oxo-pregn-4-en-3β-yl Hemisuccinate **4**

Compound **4** (166 mg, 40%) was prepared by *General Procedure I* from 3β-hydroxy-pregn-4-en-20-one **26** (316.5 mg, 1.0 mmol) by column chromatography (10–20% ethyl acetate in petroleum ether): mp 172–174 °C (EtOAc-*n*-heptane); [α]_D_^20^ 64.3 (_c_ 0.213, CHCl_3_). ^1^H NMR (400 MHz, CDCl_3_): δ 0.63 (s, 3H, H-18), 1.06 (3H, s, H-19), 2.11 (3H, s, H-21), 2.52 (1H, t, *J* = 8.9, H-17), 2.65 (4H, m, H-2′ and H-3′), 5.22 (1H, m, H-4), 5.26 (1H, m, H-3). ^13^C NMR (101 MHz, CDCl_3_): δ 209.80, 177.10, 172.18, 149.41, 119.17, 71.49, 63.82, 56.43, 54.18, 44.24, 38.99, 37.45, 35.99, 35.06, 32.98, 32.23, 31.67, 29.39, 28.98, 25.12, 24.54, 22.94, 21.13, 18.95, 13.51. IR (CHCl_3_): 1717, 1700 (C=O); 1659 (C=C); 1184, 1111 (C–O). MS ESI *m*/*z*: 415.3 (100%, M – 1), 416.3 (30%, M). HR-MS (ESI) *m*/*z* for C_25_H_35_O_5_ [M – 1] calcd 415.24900, found 415.24879. For C_25_H_36_O_5_ (416.6) calcd: 72.08%, C; 8.71% H. Found: 72.29%, C; 8.63%, H.

##### 20-Oxo-pregn-4-en-3β-yl Hemiglutarate **5**

Compound **5** (265 mg, 62%) was prepared by *General Procedure I* from 3β-hydroxy-pregn-4-en-20-one **26** (316.5 mg, 1.0 mmol) by column chromatography (15–30% ethyl acetate in petroleum ether): mp 138–140 °C (EtOAc-*n*-heptane); [α]_D_^20^ 67.4 (*c* 0.224, CHCl_3_). ^1^H NMR (400 MHz, CDCl_3_): δ 0.63 (3H, s, H-18), 1.06 (3H, s, H-19), 1.96 (2H, p, *J* = 7.3, H-3′), 2.11(3H, s, H-21), 2.39 (2H, t, *J* = 7.3, H-2′), 2.44 (2H, t, *J* = 7.3, H-4′), 2.52 (1H, t, *J* = 8.9, H-17), 5.21 (1H, m, H-4), 5.24 (1H, m, H-3). ^13^C NMR (101 MHz, CDCl_3_): δ 209.81, 177.99, 172.89, 149.32, 119.33, 71.08, 63.82, 56.43, 54.20, 44.24, 38.99, 37.45, 35.99, 35.12, 33.68, 32.98, 32.96, 32.25, 31.67, 25.22, 24.54, 22.93, 21.12, 20.05, 18.95, 13.51. IR (CHCl_3_): 1722, 1711 (C=O); 1661 (C=C); 1189, 1111 (C–O). MS ESI *m*/*z*: 429.3 (100%, M – 1), 430.3 (35%, M). HR-MS (ESI) *m*/*z* for C_26_H_37_O_5_ [M – 1] calcd 429.26465, found 429.26446. For C_26_H_38_O_5_ (430.6) calcd: 72.53%, C; 8.90% H. Found: 72.34%, C; 8.79%, H.

##### 20-Oxo-pregn-4-en-3β-yl Hemiadipate **6**

Compound **6** (231 mg, 51%) was prepared by *General Procedure II* from 3β-hydroxy-pregn-4-en-20-one **26** (318.5 mg, 1.0 mmol) by column chromatography (15–30% ethyl acetate in petroleum ether): mp 91–93 °C (EtOAc-*n*-heptane); [α]_D_^20^ 60.7 (*c* 0.201, CHCl_3_). ^1^H NMR (400 MHz, CDCl_3_): δ 0.63 (3H, s, H-18), 1.06 (3H, s, H-19), 2.11 (3H, s, H-21), 2.33 (2H, m, H-2′), 2.38 (2H, m, H-5′), 2.52 (1H, t, *J* = 8.9, H-17), 5.22 (1H, m, H-4), 5.24 (1H, m, H-3). ^13^C NMR (101 MHz, CDCl_3_): δ 209.80, 178.39, 173.32, 149.24, 119.42, 70.92, 63.82, 56.43, 54.22, 44.24, 39.00, 37.45, 36.00, 35.14, 34.39, 33.58, 32.98, 32.25, 31.67, 25.23, 24.54, 24.51, 24.24, 22.93, 21.12, 18.94, 13.51. IR (CHCl_3_): 1750, 1720, 1709 (C=O); 1661 (C=C); 1183, 1111 (C–O). MS ESI *m*/*z*: 443.3 (100%, M – 1), 444.3 (34%, M). HR-MS (ESI) *m*/*z* for C_27_H_39_O_5_ [M – 1] calcd 443.28030, found 443.28004. For C_27_H_40_O_5_ (444.6) calcd: 72.94%, C; 9.07% H. Found: 72.56%, C; 8.82%, H.

##### 20-Oxo-pregn-5-en-3β-yl Hemioxalate **7**

Following the literature, compound **7** was prepared from pregnenolone.^34^

##### 20-Oxo-pregn-5-en-3β-yl Hemimalonate **8**

Following the literature, compound **8** was prepared from pregnenolone.^34^

##### 20-Oxo-pregn-5-en-3β-yl Hemisuccinate **9**

Following the literature, compound **9** was prepared from pregnenolone.^34^

##### 20-Oxo-pregn-5-en-3β-yl Hemiglutarate **10**

Following the literature, compound **10** was prepared from pregnenolone.^34^

##### 20-Oxo-pregn-5-en-3β-yl Hemiadipate **11**

Following the literature, compound **11** was prepared from pregnenolone.^34^

##### 20-Oxo-pregn-5-en-3β-yl Hemipimelate **12**

Following the literature, compound **12** was prepared from pregnenolone.^34^

##### 20-Oxo-pregn-5-en-3β-yl Hemisuberate **13**

Following the literature, compound **13** was prepared from pregnenolone.^34^

##### Pregn-5-en-3β-yl Hemisuccinate **14**

Following the literature, compound **14** was prepared from 3β-hydroxy-pregn-5-en-20-one.^34^

##### Pregn-5-en-3β-yl Hemiglutarate **15**

Following the literature, compound **15** was prepared from 3β-hydroxy-pregn-5-en-20-one.^34^

##### Pregn-5-en-3β-yl Hemiadipate **16**

Following the literature, compound **16** was prepared from 3β-hydroxy-pregn-5-en-20-one.^34^

##### 17-Oxo-androst-5-en-3β-yl Hemiglutarate **17**

Following the literature, compound **17** was prepared from dehydroepiandrosterone.^34^

##### 17-Oxo-androst-5-en-3β-yl Hemiadipate **18**

Following the literature, compound **18** was prepared from dehydroepiandrosterone.^34^

##### 17-Oxo-androst-5-en-3β-yl Hemipimelate **19**

Following the literature, compound **19** was prepared from dehydroepiandrosterone.^34^

##### 17-Oxo-androst-5-en-3β-yl Hemisuberate **20**

Following the literature, compound **20** was prepared from dehydroepiandrosterone.^34^

##### Androst-5-en-3β-yl Hemisuccinate **21**

Following the literature, compound **21** was prepared from androst-5-en-3β-ol.^34^

##### Androst-5-en-3β-yl Hemiglutarate **22**

Following the literature, compound **22** was prepared from androst-5-en-3β-ol.^34^

##### Androst-5-en-3β-yl Hemiadipate **23**

Following the literature, compound **23** was prepared from androst-5-en-3β-ol.^34^

##### Androst-5-en-3β-yl Hemipimelate **24**

Following the literature, compound **24** was prepared from androst-5-en-3β-ol.^34^

##### 3β-Hydroxy-5α-pregnan-20-one **25**

Following the literature, compound **25** was prepared from pregnenolone.^26,27^

##### 3β-Hydroxy-pregn-4-en-20-one **26**

Compound **26** was prepared according to the literature.^28^

Sodium borohydride (0.189 g, 5 mmol, 0.5 equiv) was then added in bulk to a solution of progesterone (3.14 g, 10.0 mmol) and cerium chloride heptahydrate (3.73 g, 10.0 mmol, 1 equiv) in methanol (100 mL) under argon at −20 °C. Then, the temperature was allowed to attain −16 °C and the mixture was stirred for 15 min. Then, acetone (37 mL) was added, and the solution was allowed to reach room temperature. After the addition of water (25 mL), the solvent volume was reduced by approximately 100 mL. The product was dissolved with ether–water mixture, which caused the solution to become clear and colorless. The aqueous layer was extracted with ether (3 × 30 mL). The combined organic phase was washed with brine and dried, and solvents were evaporated. The crude material was purified by column chromatography (20–25% ethyl acetate in petroleum ether) affording 1.57 g (50%) of **26**. The purity and identity of compound **26** were confirmed by ^1^H NMR, which was identical with the literature. ^1^H NMR (400 MHz, CDCl_3_): δ 0.63 (s, 3H, s, H-18), 1.05 (3H, s, H-19), 2.11 (3H, s, H-21), 2.51 (1H, t, *J* = 9.0, H-17), 4.12–4.18 (1H, m, H-3), 5.29 (1H, d, *J* = 1.8, H-4). Therefore, compound **26** was used in further synthesis without further characterization.

### Biology

#### Chemicals

17β-estradiol, NAD^+^, benzonase, lysozyme, and anhydrous DMSO were purchased from Sigma-Aldrich (Prague, Czech Republic). cOmplete EDTA-free protease inhibitor cocktail was purchased from Roche. Allopregnanolone was purchased from Tocris Bioscience.

#### Production and Purification of Human Recombinant

17β-HSD10 was performed as described previously.^11^ Briefly, the autoinduction and overexpression were performed at 25 °C for 18 h in the *E. coli* BL21 (DE3) strain. The bacterial pellet was resuspended in lysis buffer that consisted of sodium phosphate buffer (50 nM), NaCl (150 mM), and imidazole (10 mM, pH 8.0) with 1 mg/mL lysozyme and benzonase (150 U/mL). cOmplete EDTA-free protease inhibitor cocktail was incubated for 20 min on ice and then sonicated (12 × 10 s pulses with 30 s pauses). The supernatant was applied to the Ni-NTA agarose resin after centrifugation (16 000*g*, 10 min, 4 °C). Then, the solution was incubated on ice for 1 h with gentle stirring.

The resin was washed three times with 10 mL of washing buffer I that consisted of sodium phosphate buffer (50 nM), NaCl (150 mM), and imidazole (10 mM, pH 8.0). Then, the resin was washed by 3 × 10 mL of washing buffer II that consisted of sodium phosphate buffer (50 nM), NaCl (150 mM), and imidazole (40 mM, pH 8.0). Elution was performed using elution buffer that consisted of sodium phosphate buffer (50 nM), NaCl (150 mM), and imidazole (250 mM, pH 8.0). The elution buffer was exchanged for storage buffer [71 mM Tris-HCl, 214 mM NaCl (pH 8.0)] using an Amicon Ultra-4 Centrifugal Filter Unit (10 000 MWCO). The enzyme was stored at −80 °C. The protein concentration was measured using the Bradford assay, and the purity of 17β-HSD10 was confirmed by SDS-PAGE.

#### Inhibition of 17β-Estradiol to Estrone Transformation

The standard steroids were screened at 1 and 10 μM concentrations, and the tested compounds and standard AG18051 were screened at 10 μM concentration. For compounds with at least 50% inhibition of 17β-HSD10, the IC_50_ values were determined.

Steroid stock solutions (5 mM) were further diluted with DMSO to the working concentrations. DMSO was used as a vehicle control, and AG18051 was used as a control inhibitor. The enzyme activity was determined fluorometrically at 37 °C in the activity assay buffer [100 mM potassium phosphate buffer (pH 8.0)]. The general reaction mixture (200 μL per well) consisted of 17β-estradiol (25 μM, DMSO), NAD+ (500 μM, deionized water), recombinant 17β-HSD10 (45 nM), and different concentrations of inhibitor (2.2% (v/v) final concentration of DMSO as solvent). Before the addition of substrate, the reaction mixture of enzyme, cofactor, and inhibitor in the assay buffer was preincubated for 5 min at 37 °C. The increase of fluorescence (Ex/Em = 340/460 nm) due to the formation of NADH was monitored for 20 min (30 s intervals). For IC_50_ value determination, dose–response inhibition at 11 different concentrations of inhibitor (0.6–33.75 μM) was determined and the data were analyzed by GraphPad Prism 8.4.3 (GraphPad Software Inc.). All measurements were performed in a tetraplicate.

#### Inhibition of Allopregnanolone to 5α-Dihydroprogesterone Transformation

Compound **23** (5 mM stock solution) was diluted with DMSO to the working concentrations. DMSO was used as vehicle control. The enzyme activity was determined as described in the previous section; the only difference was the replacement of 25 μM 17β-estradiol with 20 μM allopregnanolone as 17β-HSD10 substrate. For IC_50_ value determination, dose–response inhibition at 10 different concentrations of inhibitor (0.16–33.75 μM) was determined, and the data were analyzed by GraphPad Prism 8.4.3 (GraphPad Software Inc.). All measurements were performed in a tetraplicate.

#### Inhibition Type Determination

The type of inhibition of compound **23** was determined with respect to the substrate 17β-estradiol. The inhibitor was determined at three different concentrations (3, 6, and 9 μM) in combination with different concentrations of 17β-estradiol (12.5–75 μM) and a saturated NAD^+^ concentration (500 μM). As vehicle control, DMSO was used. The obtained data were analyzed using GraphPad Prism 8.4.3 (GraphPad Software Inc.). All measurements were performed in triplicate.

#### Luminescent Cell Viability Assay and CellTox Green Cytotoxicity Assay

HEK-293 cells in DMEM (Capricorn) were supplemented with fetal bovine serum (10%, Gibco), l-glutamine (2 mM, Lonza), and nonessential amino acid additives (Gibco). Cells were maintained at 37 °C under a humidified atmosphere of 5% CO_2_. Compound **23** was tested on the HEK-293 cell line on cell viability using CellTox Green (G8741, Promega) and the CellTiter-Glo 2.0 kit (G9241, Promega), respectively, to establish their cytotoxic effect. Using a Tecan Spark 10 M instrument, the measurements were performed as end point methods in the multiplex. For multiplex measurement, 7500 cells were seeded per well in 50 μL of culture media and cultured for 24 h before the addition of selected compounds. Compound **23** was used at concentrations of 1 and 10 μM (1% (v/v) total concentration of DMSO in the reaction). As a vehicle control, cells were treated with 1% DMSO. As a positive control, 100 μM valinomycin treatment was used. White solid bottom 96 well microplates were used for the measurement. First, the fluorescent CellTox Green Cytotoxicity Assay (Ex/Em 485/530 nm) was performed. Next, the CellTiter-Glo Luminescent Cell Viability Assay with an integration time of 500 ms was determined.

#### Fluorogenic Assay for Cellular Inhibition of 17β-HSD10

A previously published cell line with overexpressed 17β-HSD10 (HEK-293-HSD10)^15^ was used to measure 17β-HSD10 inhibition in the cellular environment. For this purpose, the (−)–CHANA fluorogenic probe was used. The cells (density 1 × 10^4^ cells per well) were seeded in DMEM (200 μL) without phenol red (Gibco). The cells were supplemented with fetal bovine serum (10%, Gibco), l-glutamine (2 mM), nonessential amino acid additives (Gibco), and 4.5 g/L glucose (Sigma) into black clear bottom 96 well plates (Brand, 781971). The cells were incubated for 20 h following the treatment with compound **23** in DMSO/DMSO only (vehicle control). After 2 h of compound treatment, the (-)-CHANA probe was added at the final concentration of 20 μM. The changes in fluorescent intensities were measured immediately after (-)-CHANA addition and 2 h later. The fluorescence intensities of the CHANK product were taken by using the TECAN SPARK 10 M instrument (Ex/Em = 380/525 nm). The residual 17β-HSD10 activity was calculated as ΔF between 2 and 0 h after (-)-CHANA treatment, and the data were normalized between nontreated HEK-293-HSD10 and native, nontransfected HEK-293 controls (using relative response ratio). Compound **23** was used at 1, 5, 10, 15, and 25 μM concentrations to detect the ability to penetrate the cells and to influence the 17β-HSD10 enzyme activity inside the cells.

## Supporting Information Available

^1^H and ^13^C NMR spectra of compounds 1–6 (Figures S1–S12); HR-MS spectra of compounds 1–6 (Figures S13–S18); LC-MS spectra of compounds 1–6 (Figures S19– S24); *In vitro* inhibitory potency of the steroidal comparators (Table S1); Dose–response curve for compound **23** and compound **22** (Figure S25) (PDF)

## Abbreviations

17β-HSD10: 17β-hydroxysteroid dehydrogenase type 10
AG18051: (1-azepan-1-yl-2-phenyl-2-(4-thioxo-1,4-dihydropyrazolo [3–d] pyrimidin-5-yl)-ethanone)
RM-532-46: {(3α,5α,17α)-3-Hydroxy-3-[4-(3-methoxybenzyl)piperazin-1-yl]methylandrostan-17-one}
