# Correction to “C-3 Steroidal Hemiesters as Inhibitors of 17β-Hydroxysteroid Dehydrogenase Type 10”

**DOI:** 10.1021/acsomega.4c02539

**Published:** 2024-03-26

**Authors:** Michaela Hanzlova, Barbora Slavikova, Marina Morozovova, Kamil Musilek, Aneta Rotterova, Lucie Zemanová, Eva Kudova

**Affiliations:** † Faculty of Science, Department of Chemistry, University of Hradec Kralove, Rokitanskeho 62, 500 03 Hradec Kralove, Czech Republic; ‡ Institute of Organic Chemistry and Biochemistry, Czech Academy of Sciences, Flemingovo namesti 2, Prague 6 166 10, Czech Republic

The Funding statement is corrected as given below.

