# Supplementary material for: C-3 Steroidal Hemiesters as Inhibitors of 17β-Hydroxysteroid Dehydrogenase Type 10
Source: ACS Omega. 2024 Feb 28;9(10):12116–24. doi: 10.1021/acsomega.3c10148 (PMC10938439; doi:10.1021/acsomega.3c10148)
Supplement: Supplementary file 1 — ao3c10148_si_001.pdf [file ao3c10148_si_001.pdf]

Supporting information

The C-3 steroidal hemiesters as inhibitors of 17β-hydroxysteroid dehydrogenase type 10

Michaela Hanzlova[a,†], Barbora Slavikova[b], Marina Morozovova[b], Kamil Musilek[a], Aneta Rotterova[a], Lucie Zemanová[a,*], Eva Kudova[b,*]

[a]Department of Chemistry, Faculty of Science, University of Hradec Kralove, Hradec Kralove, Czech Republic

[b]Institute of Organic Chemistry and Biochemistry, Czech Academy of Sciences, Flemingovo namesti 2, Prague 6, 166 10, Czech Republic

Table of Contents

1 Chemistry 2

1.1 ^{1}H and ^{13}C NMR spectra of compounds 1-6 2

1.2 HRMS spectra of compounds 1-6 8

1.3 LC-MS analysis of compounds 1-6 11

2 *In vitro* results 21

1 Chemistry

1.1 ^{1}H and ^{13}C NMR spectra of compounds 1-6

Figure S1. ^{1}H NMR spectra of compound **1**

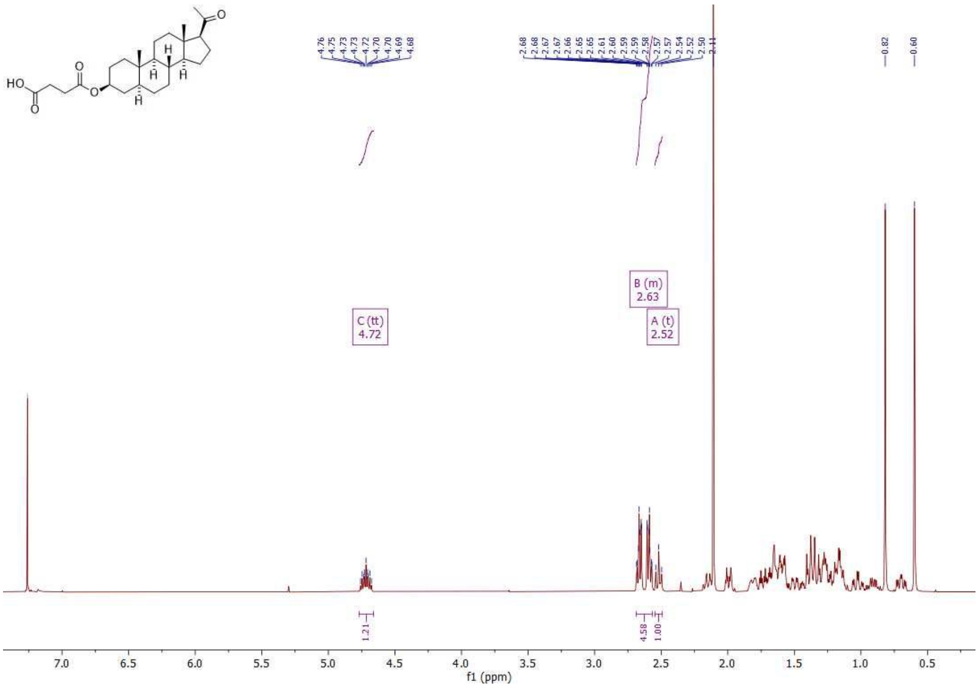

Figure S2. ^{13}C NMR spectra of compound **1**

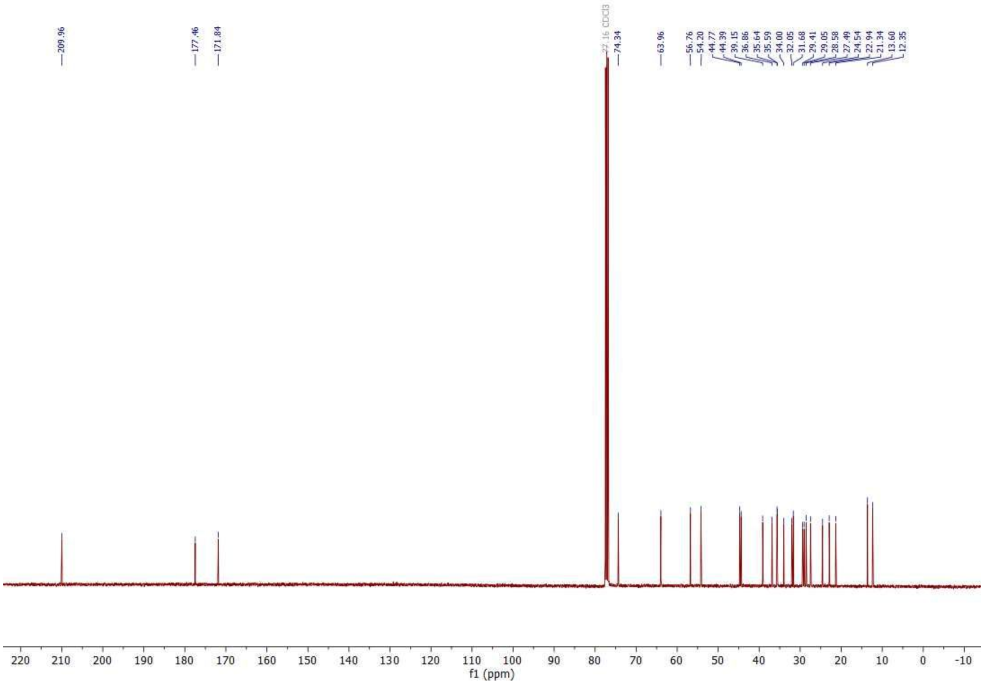

Figure S3. ^{1}H NMR spectra of compound **2**

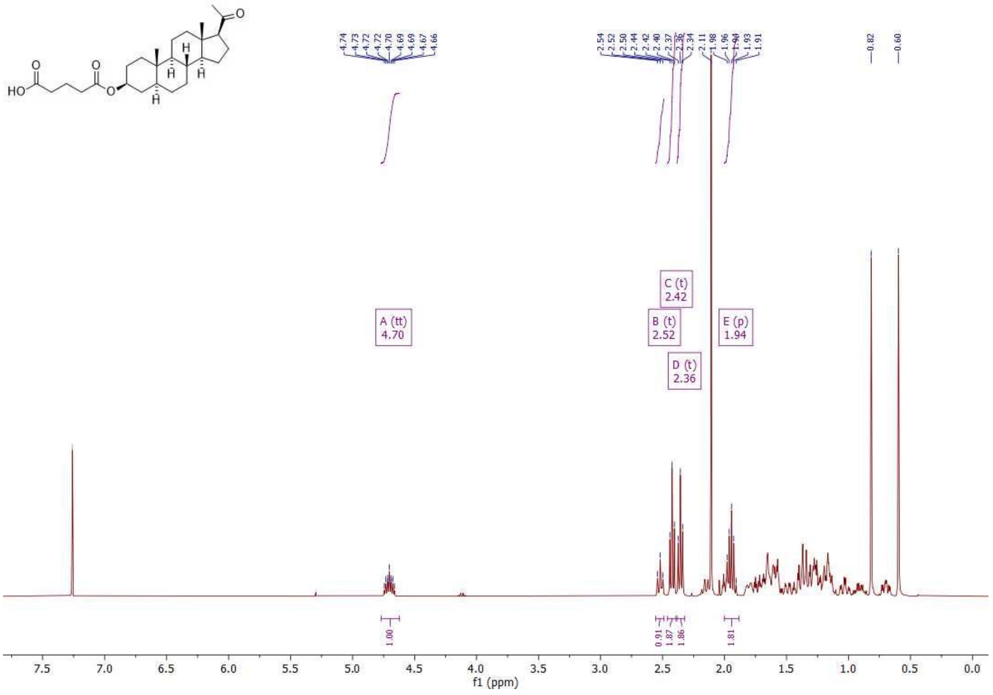

Figure S4. ^{13}C NMR spectra of compound **2**

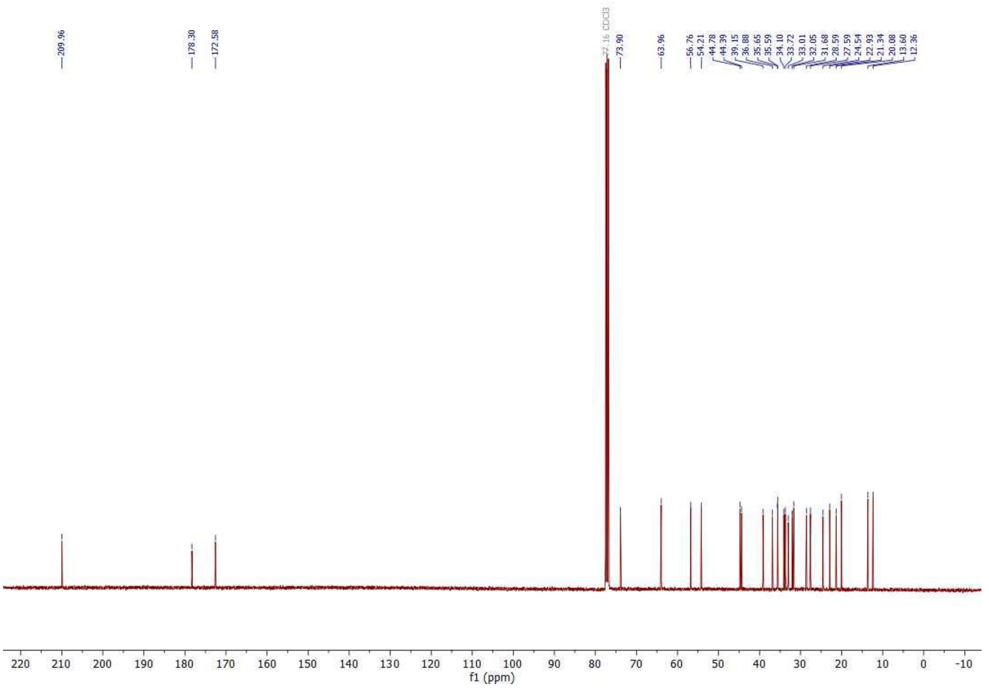

Figure S5. ^{1}H NMR spectra of compound **3**

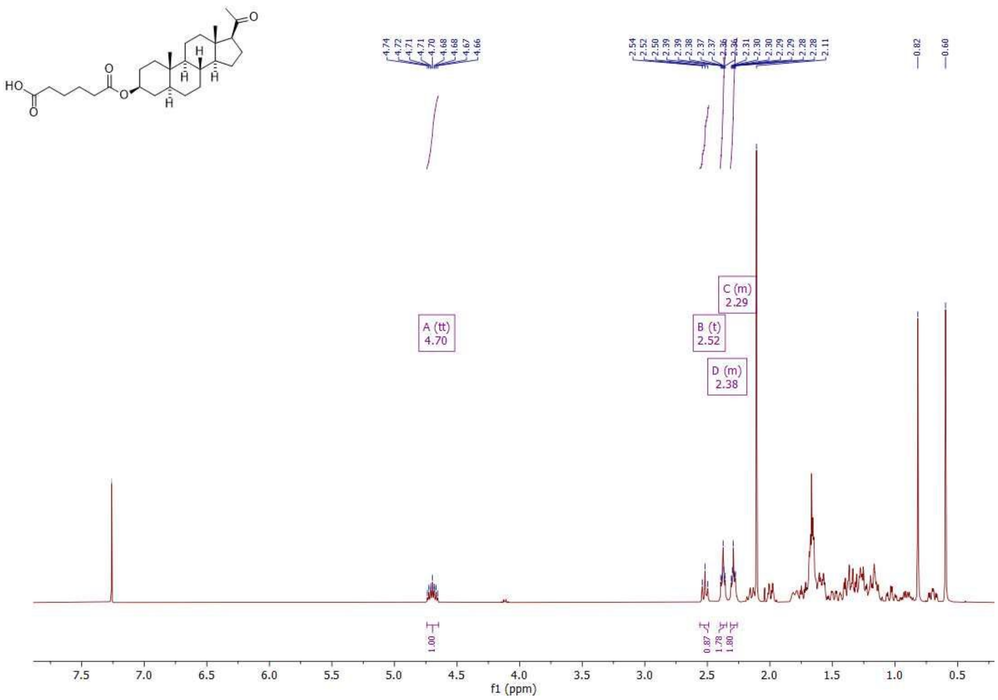

Figure S6. ^{13}C NMR spectra of compound **3**

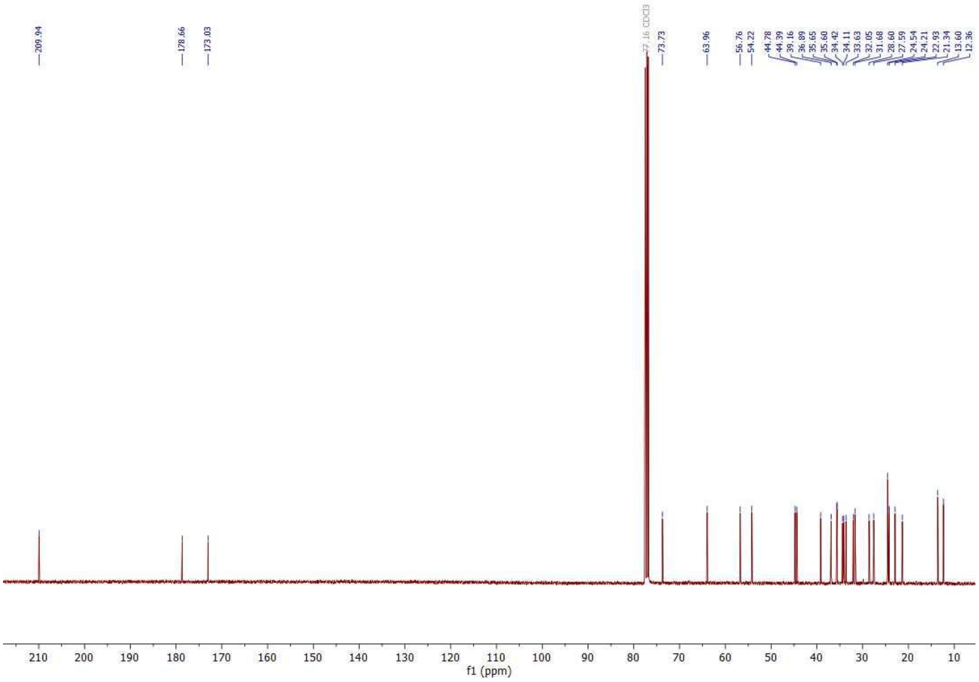

Figure S7. ^{1}H NMR spectra of compound **4**

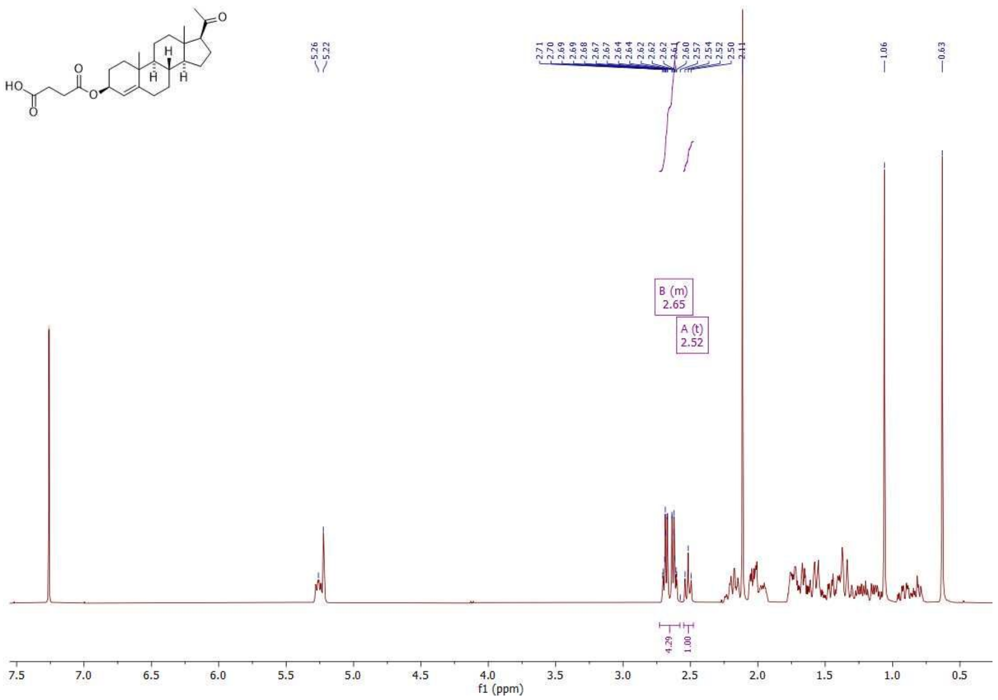

Figure S8. ^{13}C NMR spectra of compound **4**

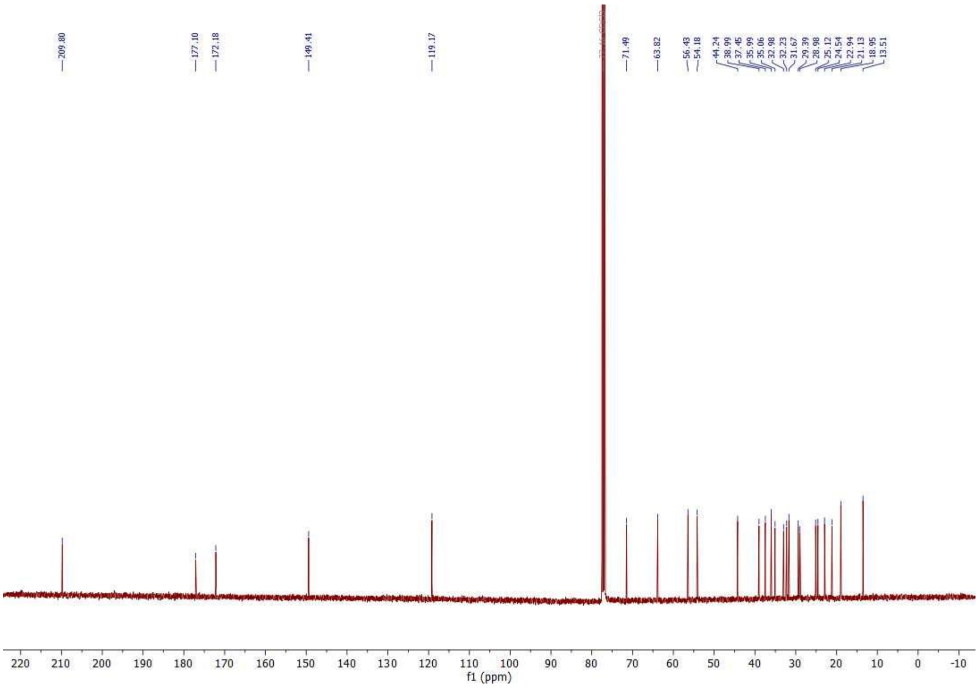

Figure S9. ^{1}H NMR spectra of compound **5**

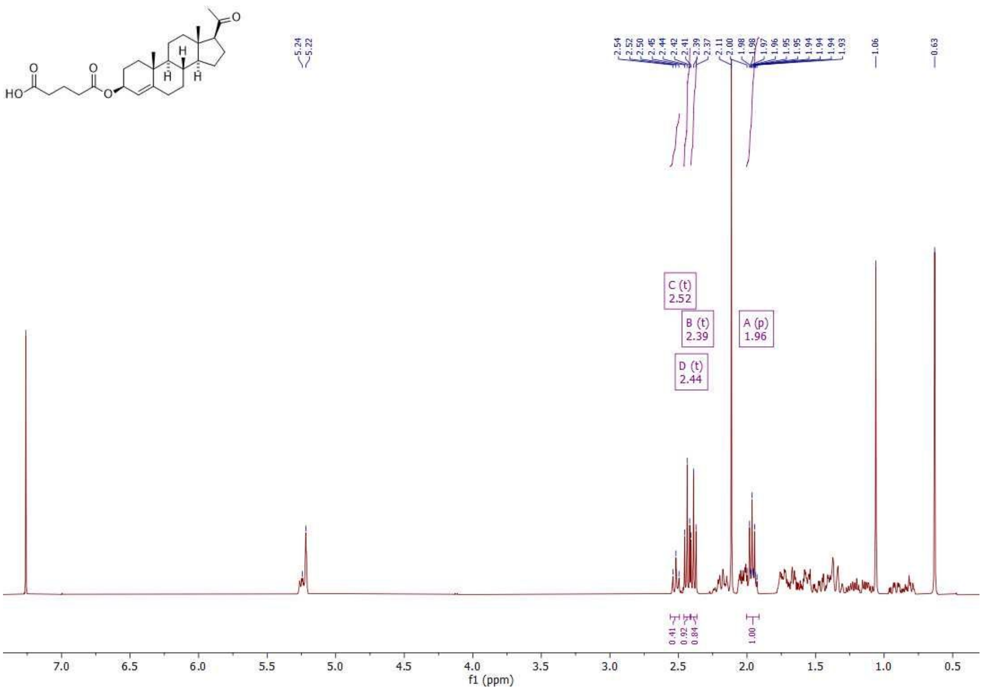

Figure S10. ^{13}C NMR spectra of compound **5**

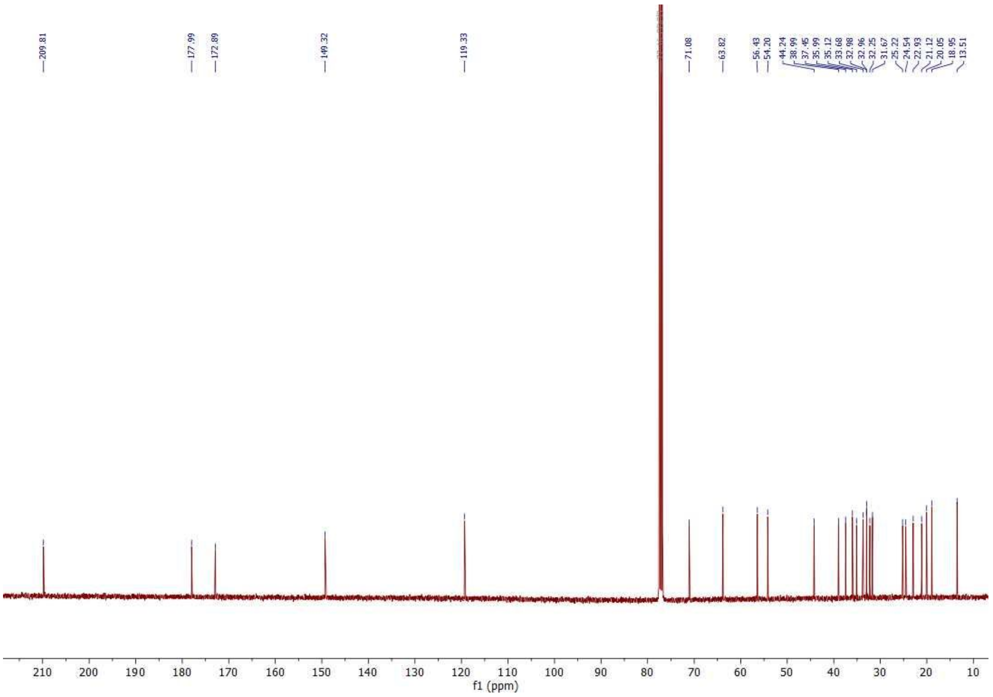

Figure S11. ^{1}H NMR spectra of compound **6**

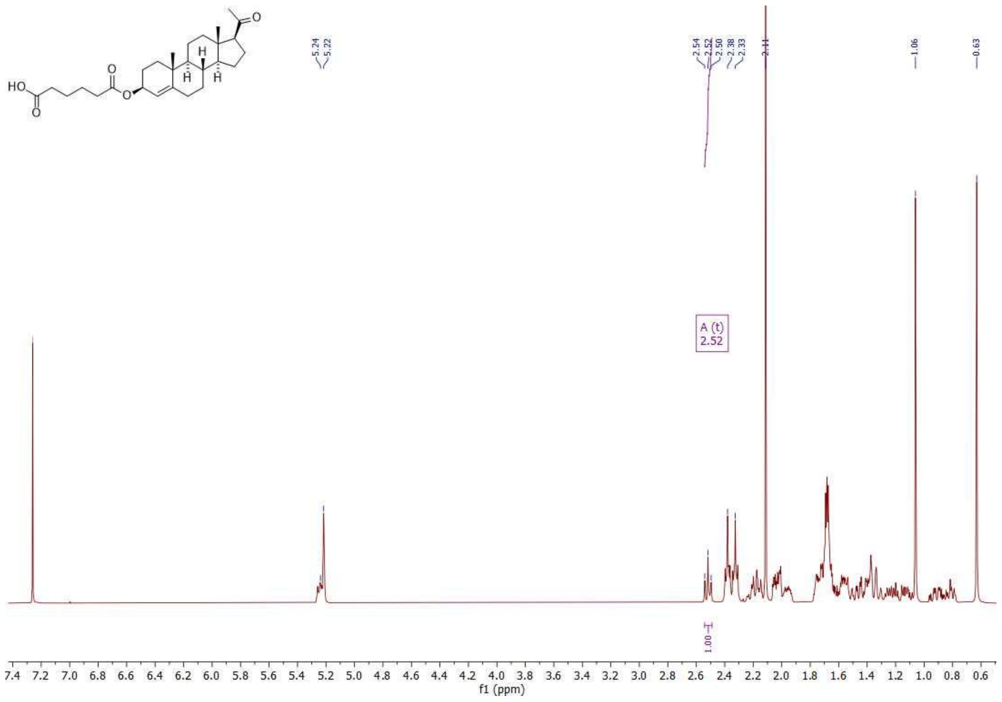

Figure S12. ^{13}C NMR spectra of compound **6**

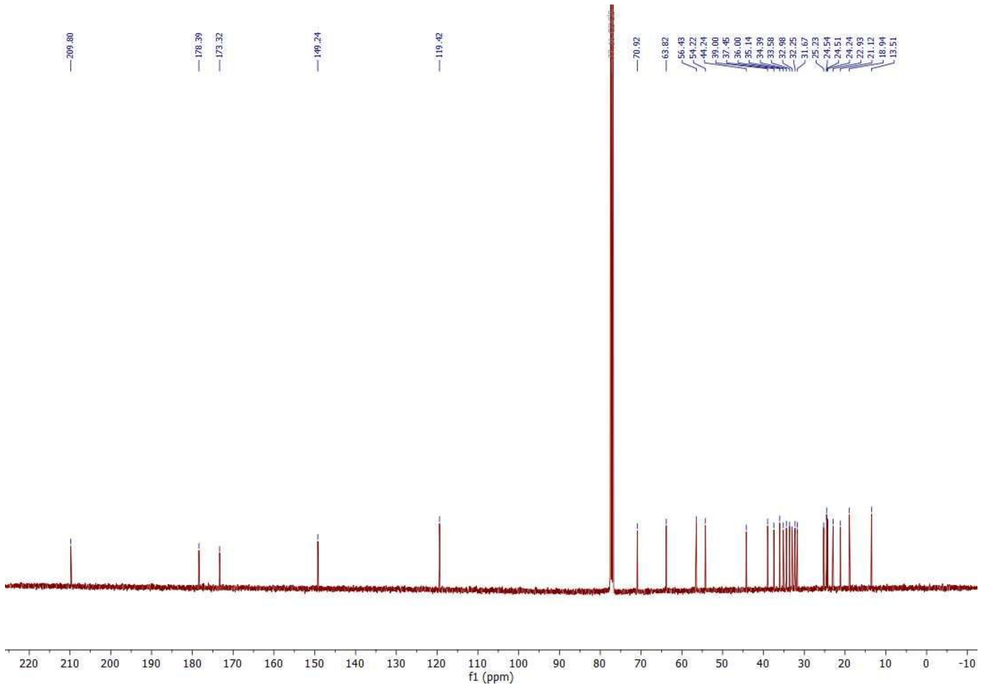

1.2 HRMS spectra of compounds 1-6

Figure S13. HRMS spectra of compound **1**

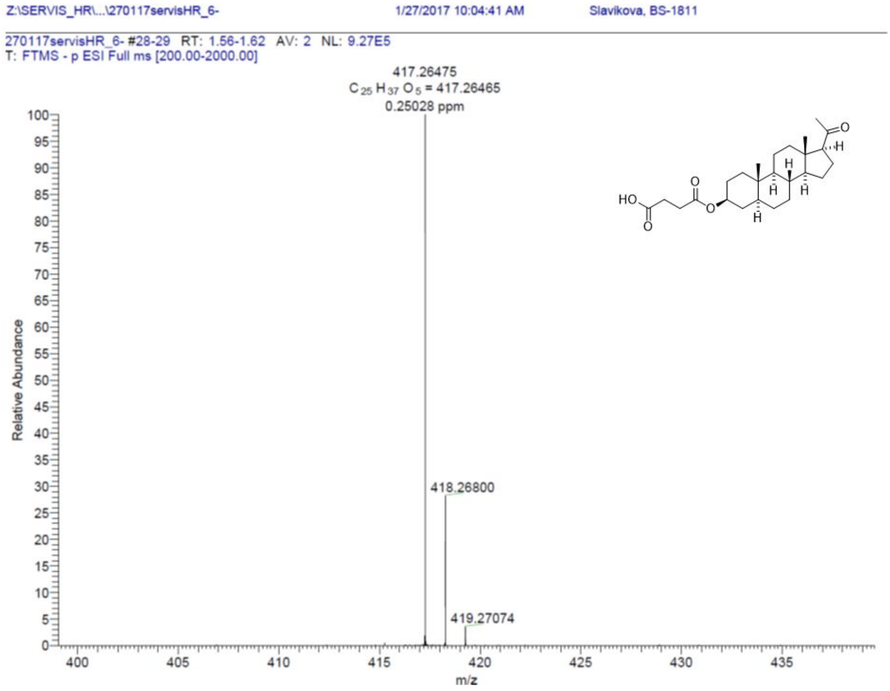

Figure S14. HRMS spectra of compound **2**

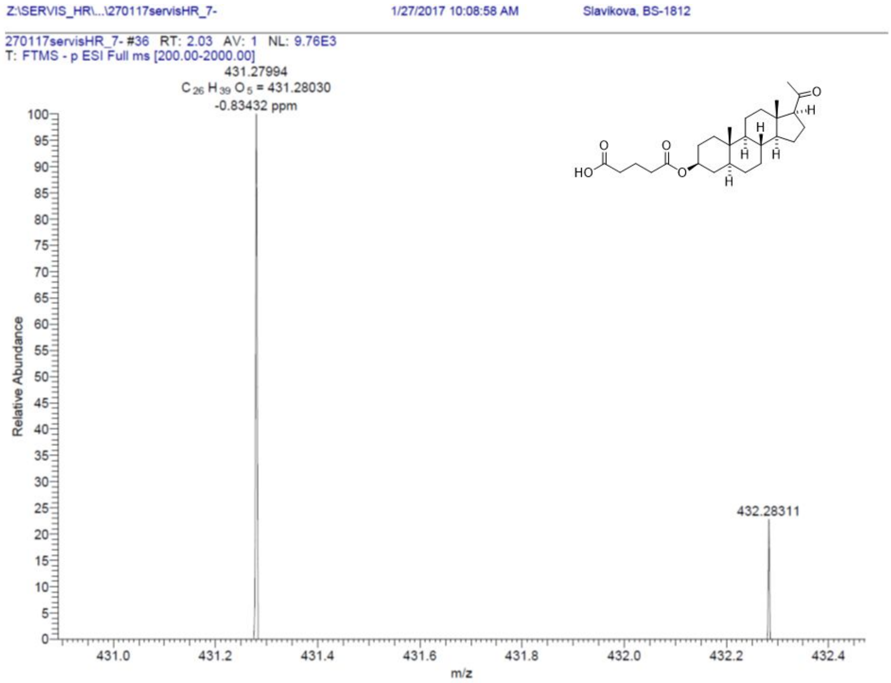

Figure S15. HRMS spectra of compound **3**

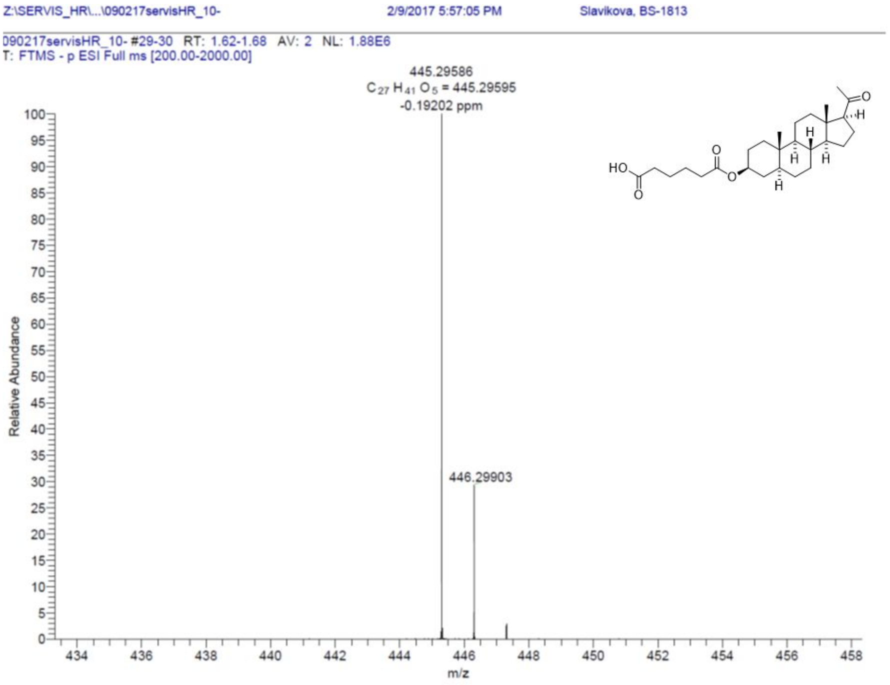

Figure S16. HRMS spectra of compound **4**

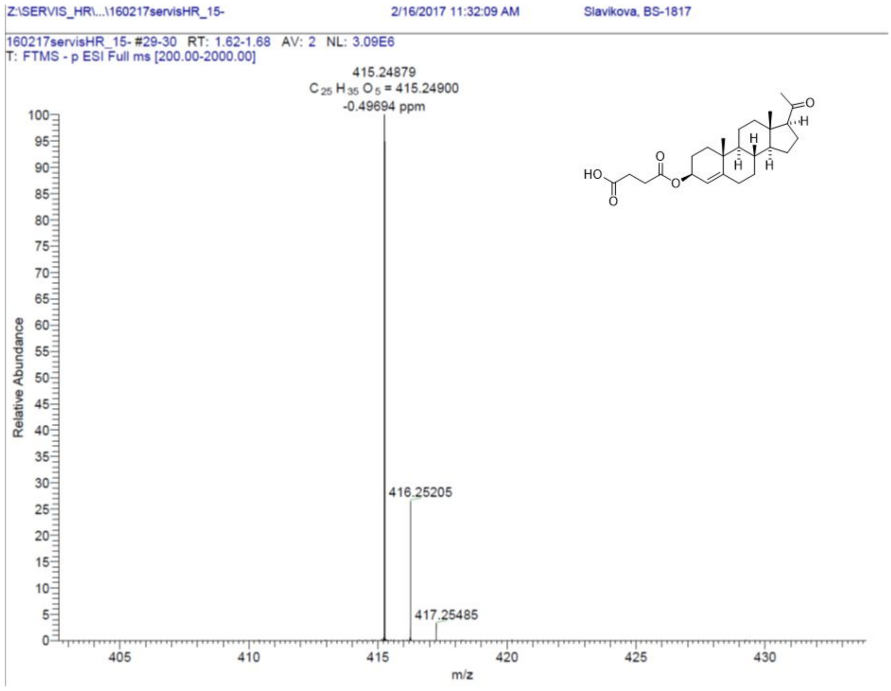

Figure S17. HRMS spectra of compound **5**

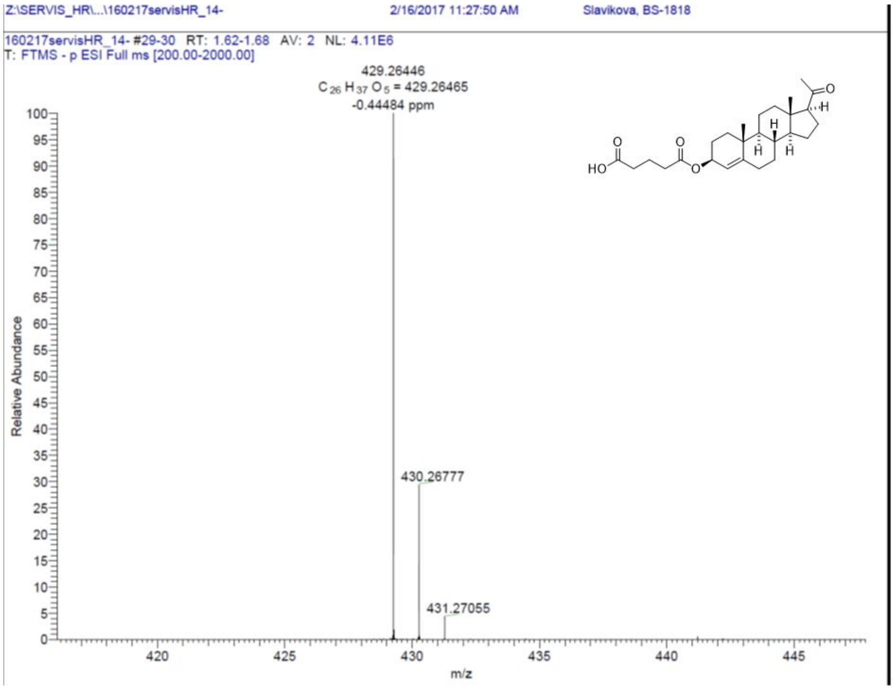

Figure S18. HRMS spectra of compound **6**

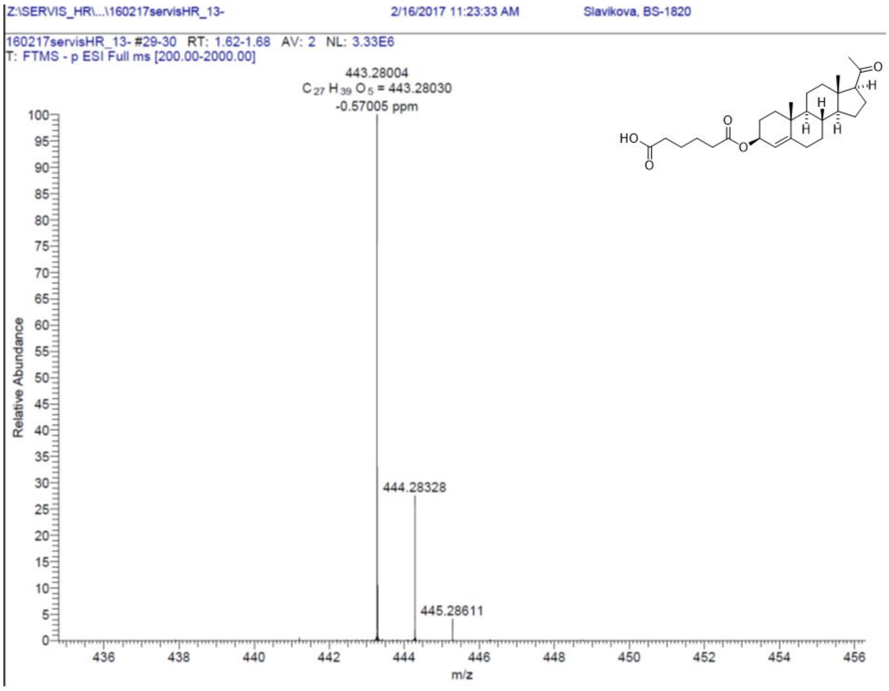

1.3 LC-MS analysis of compounds 1-6

Acetonitrile and methanol were of HPLC gradient grade, purchased from VWR Chemicals (Czech Republic) and Fisher Scientific (USA), formic acid (eluent additive for LC-MS; Fisher Scientific). Water for the HPLC mobile phase was purified by an ultrapure milli-Q water system (3P Synergy UV).

The analytical HPLC measurements were performed on Shimadzu (Tokyo, Japan) Nexera LC-40 equipped with a SCL-40 communication module, a two degasser units DGU- 403 and DGU-405, two LC-40 D X3 dual solvent delivery module, an autosampler and a thermostated column oven CTO-40S. The separation was performed using Shim-pack Scepter C8-120, 1.9 µm, 2.1x100mm (Shimadzu). The mobile phase of water/methanol/formic acid (95/5/1) (A) and acetonitrile (B) was used with isocratic elution. The flow rate of mobile phase was 0.6 ml/min , the temperature of the column was set to 40 °C, and the injection volume was 0.4 µL. An ELSD-LT II, evaporative light scattering detector (ELSD) (Shimadzu, Japan) was utilized for the analysis of the purity. The conditions for the analysis were 60 °C nebulization temperature by nitrogen and 10 the gain factor.

Mass spectrometric detection was performed on a single quadrupole LCMS-2020 (Shimadzu, Japan) with dual ion source by an electrospray ionization (ESI) probe, and simultaneously ionized by an atmospheric pressure chemical ionization (APCI) corona discharge needle located below the ESI outlet. All acquisitions were performed in both positive and negative ionization mode. The nebulizing gas had flow 1.5 L/min, the drying gas was set to 15 L/min, corona needle voltage 4500 V (for positive mode), and a heat block had temperature of 400°C. Full scan mass spectra were acquired from m/z 200-800 . The data was processing by software LabSolutions™.

Figure S19. LC-MS spectra of compound **1**

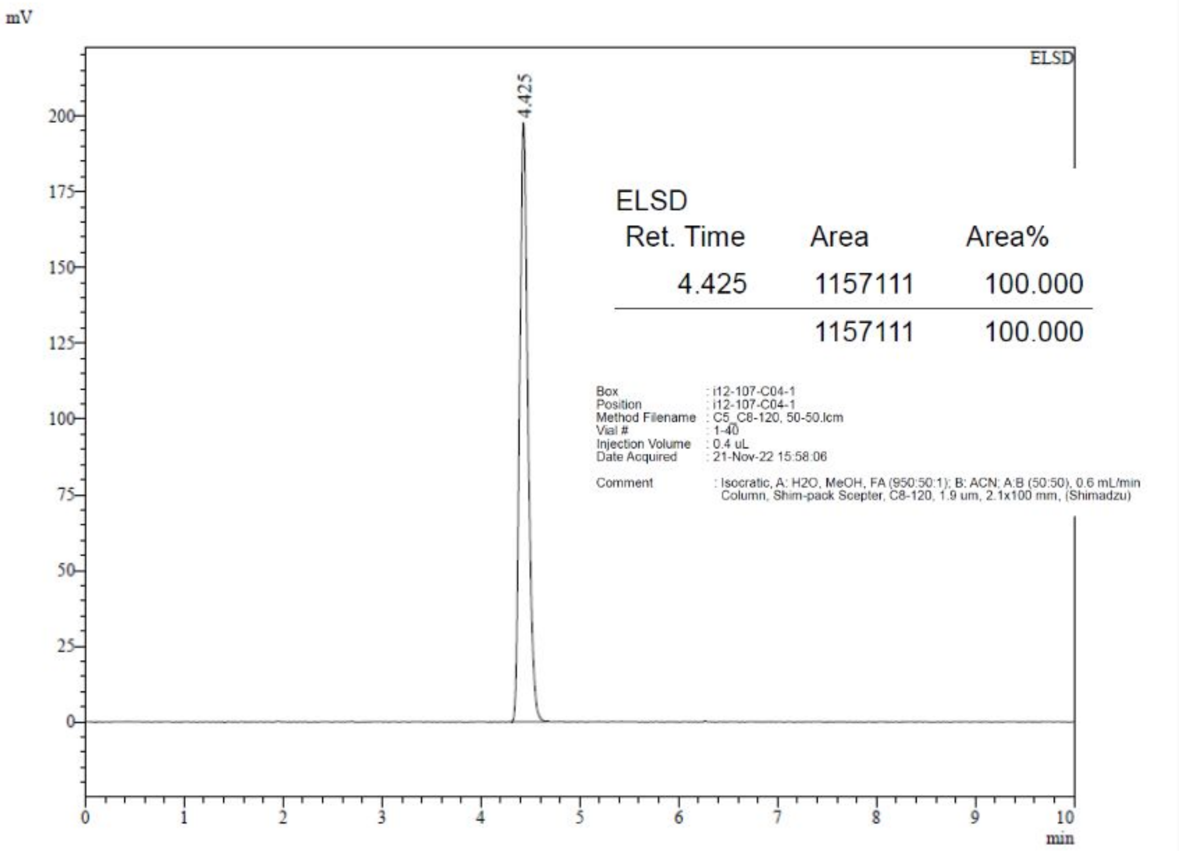

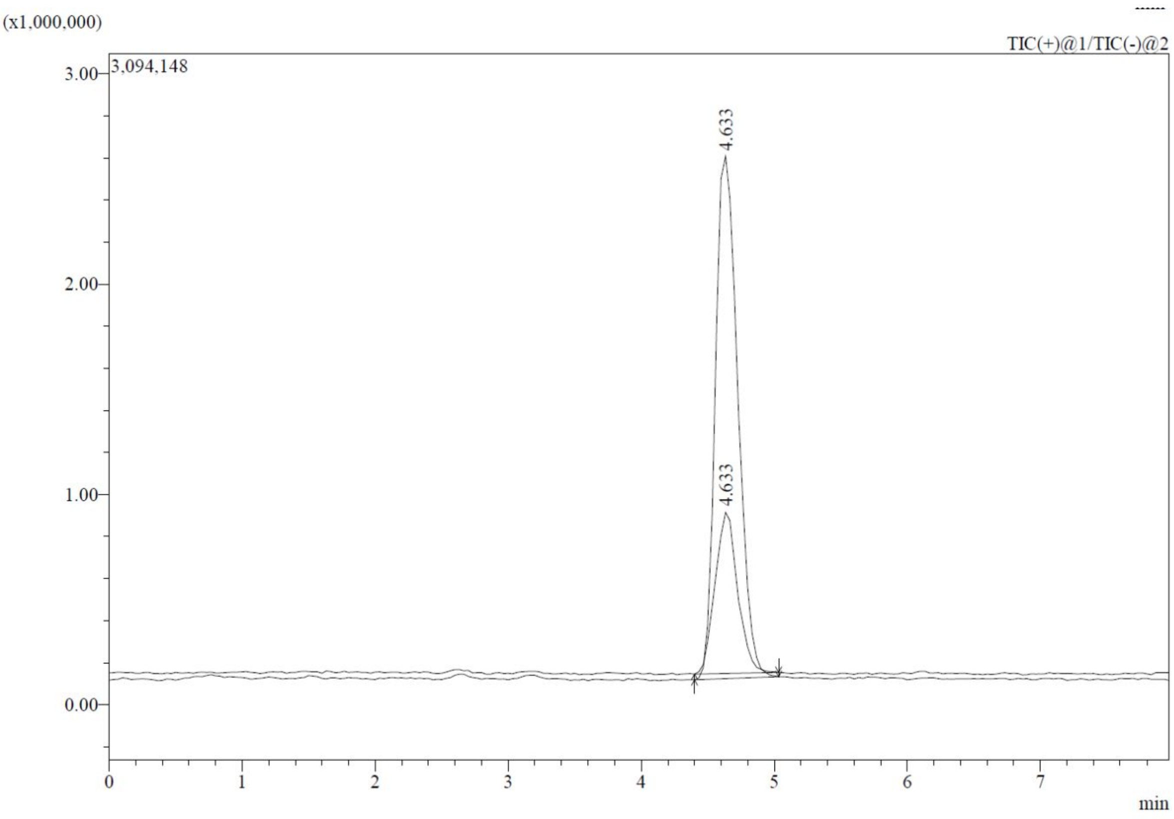

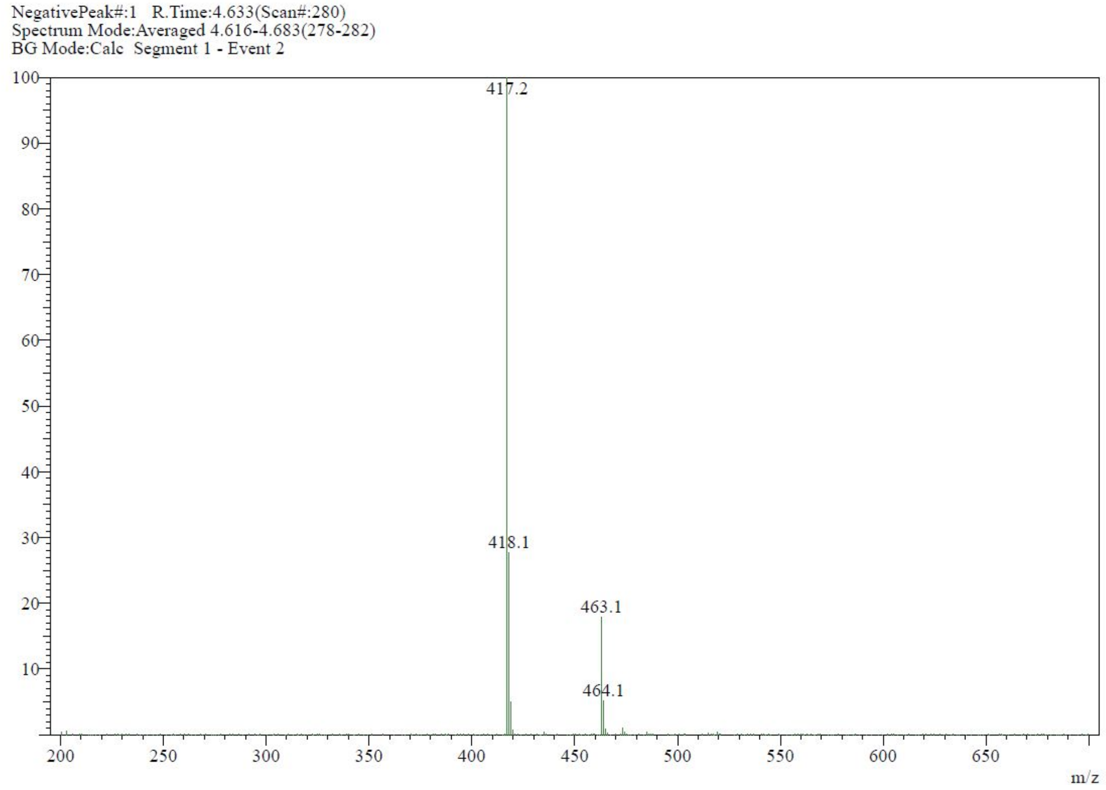

Figure S20. LC-MS spectra of compound **2**

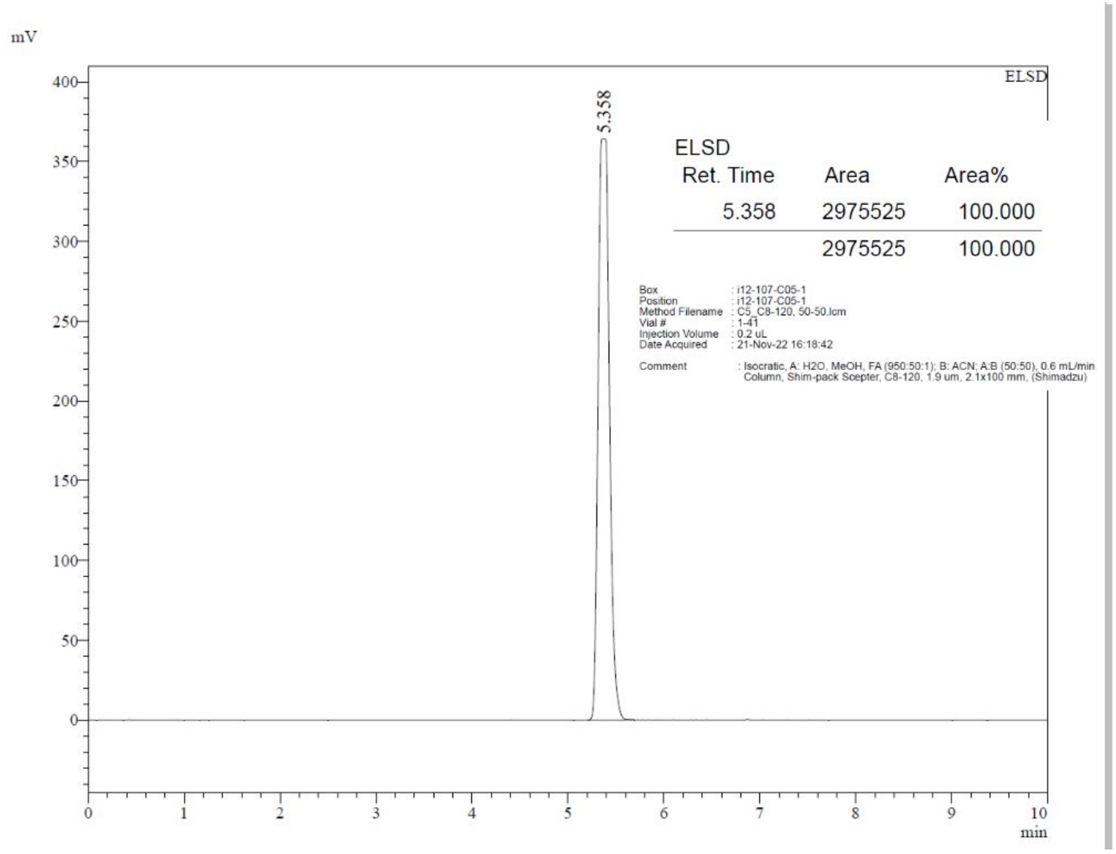

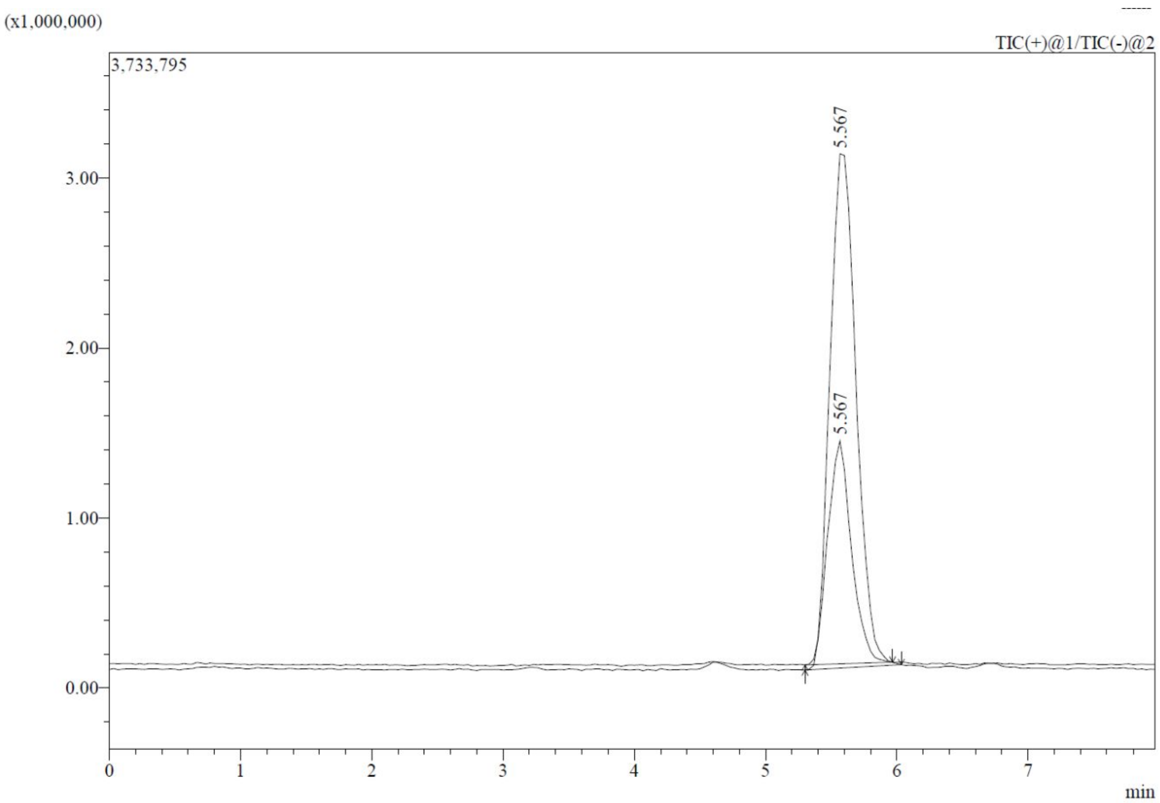

NegativePeak#:1   R.Time:5.567(Scan#:336)
Spectrum Mode:Averaged 5.550-5.616(334-338)
BG Mode:Calc Segment 1 - Event 2
Negative

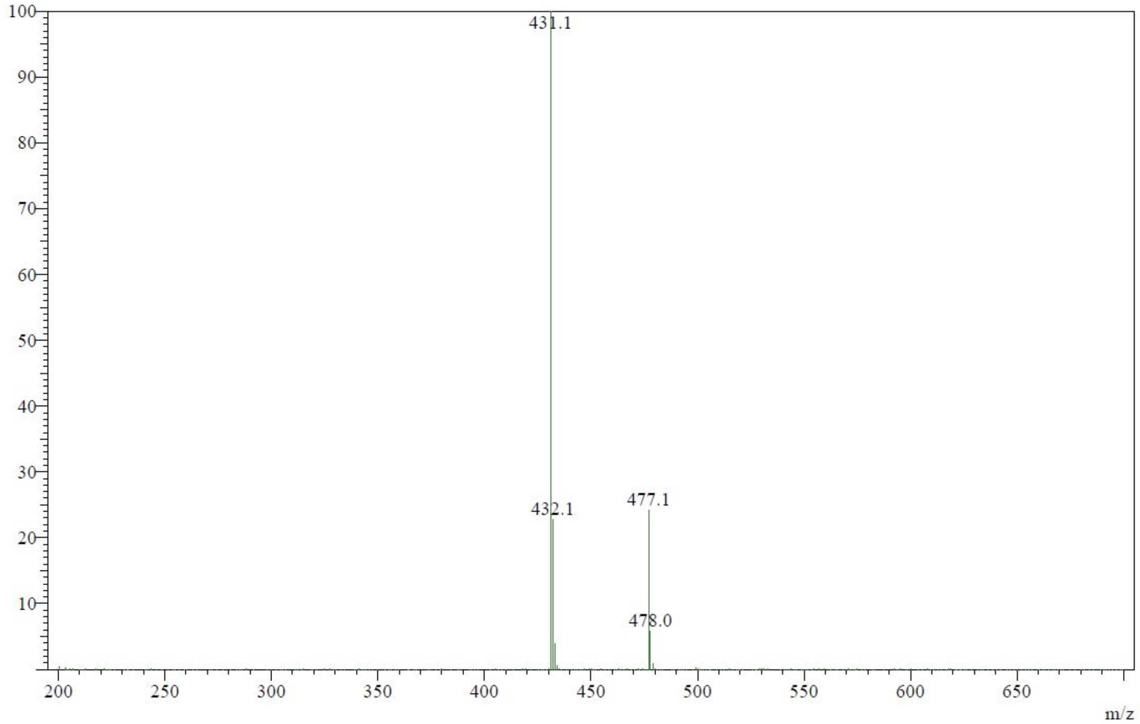

Figure S21. LC-MS spectra of compound **3**

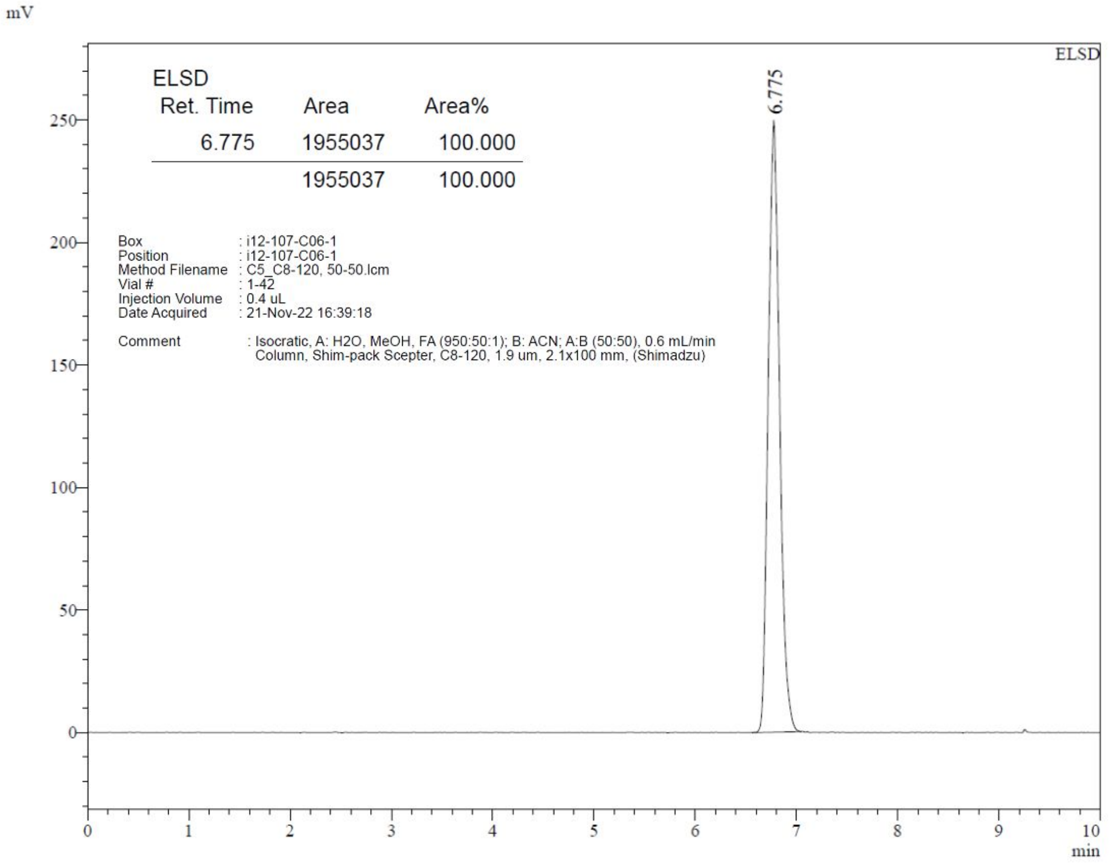

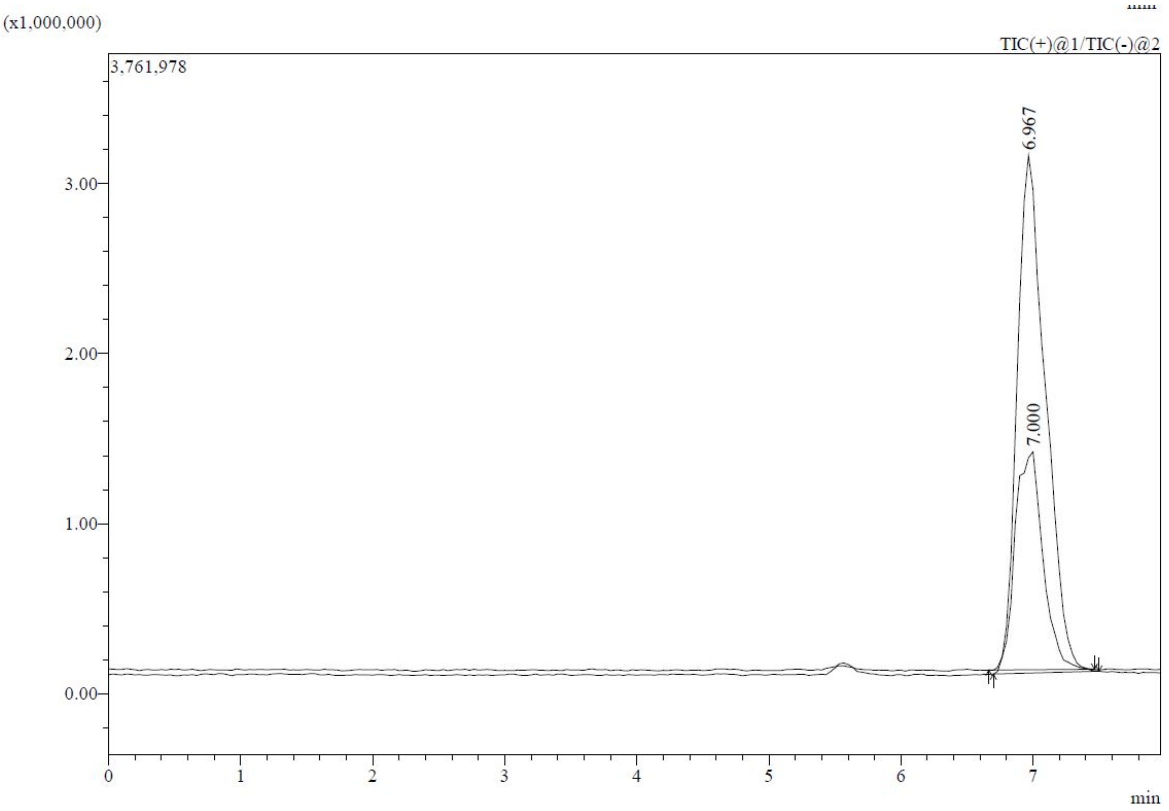

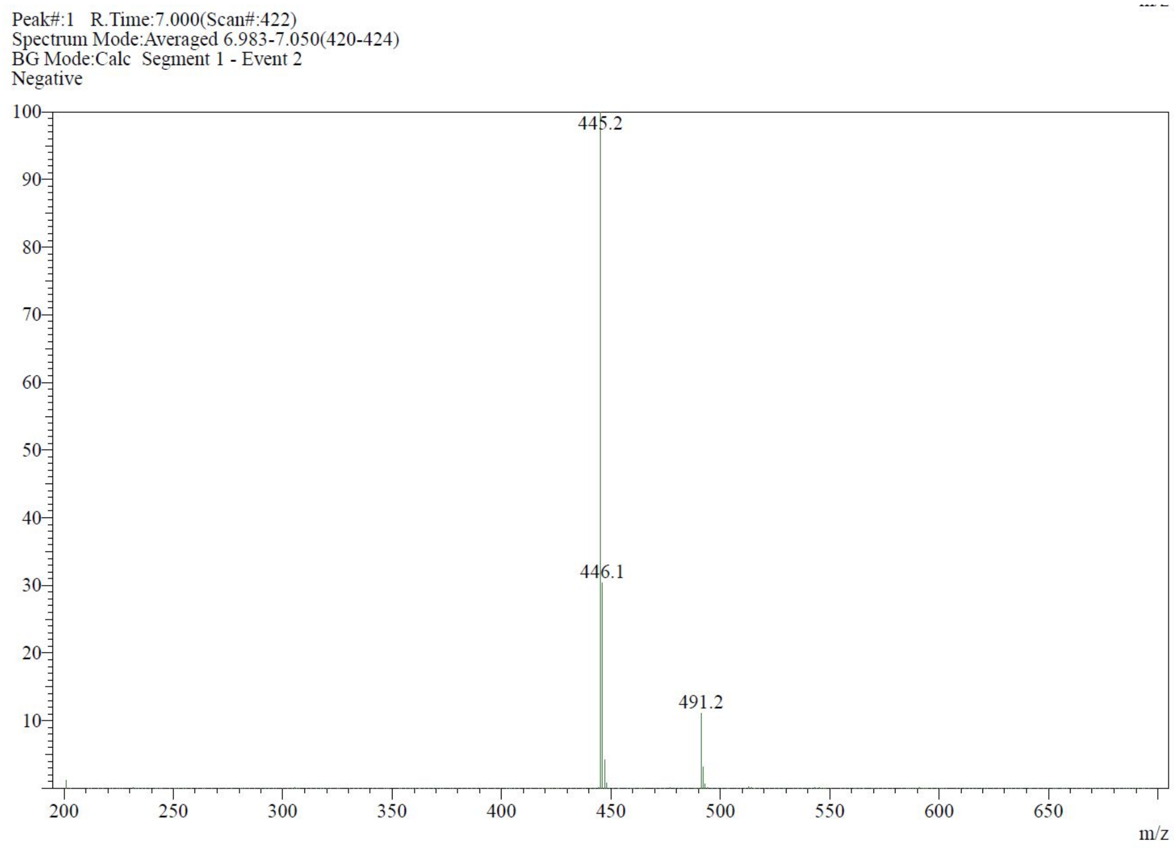

Figure S22. LC-MS spectra of compound **4**

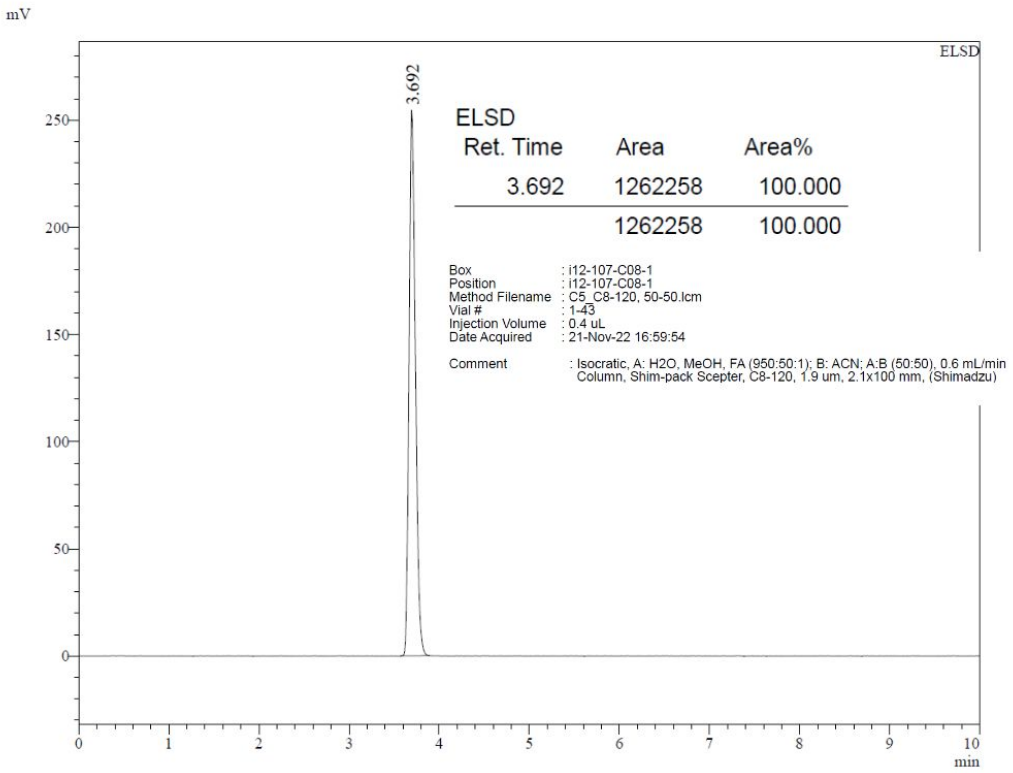

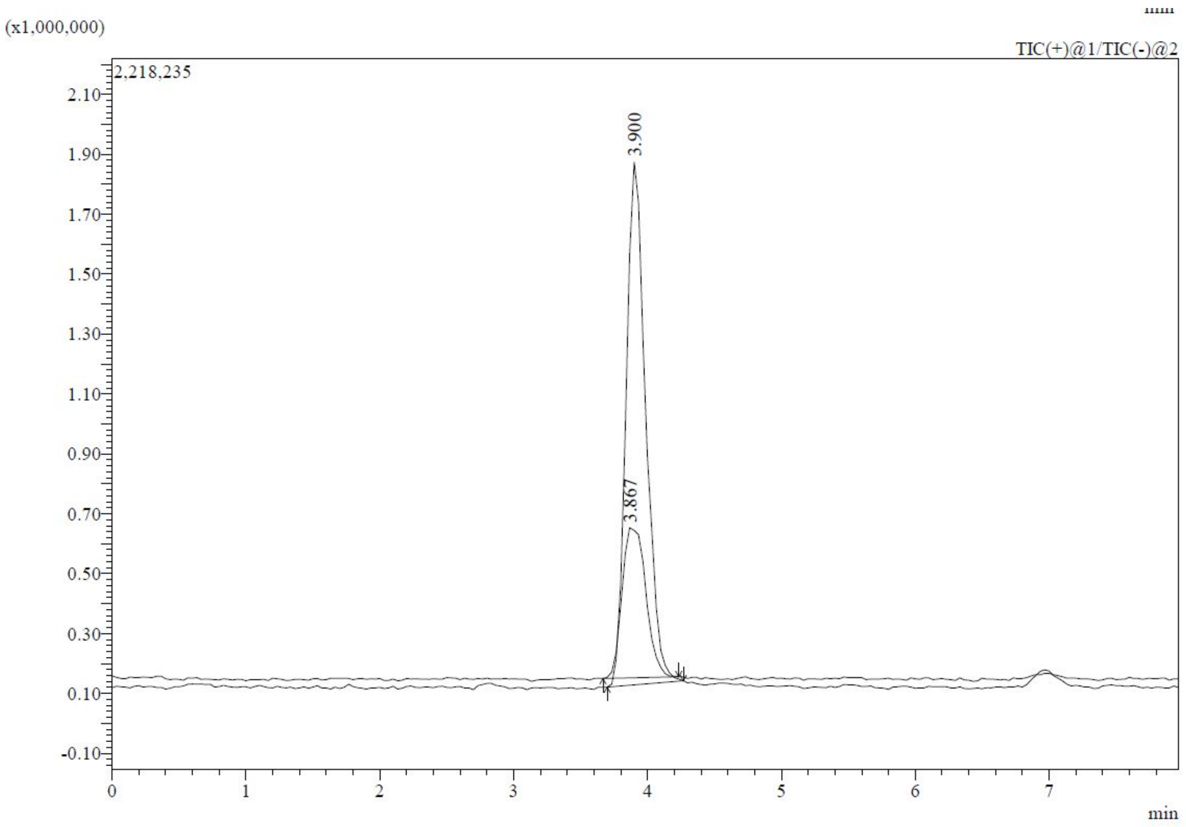

NegativePeak#:1 R.Time:3.867(Scan#:234)
Spectrum Mode:Averaged 3.850-3.916(232-236)
BG Mode:Calc Segment 1 - Event 2

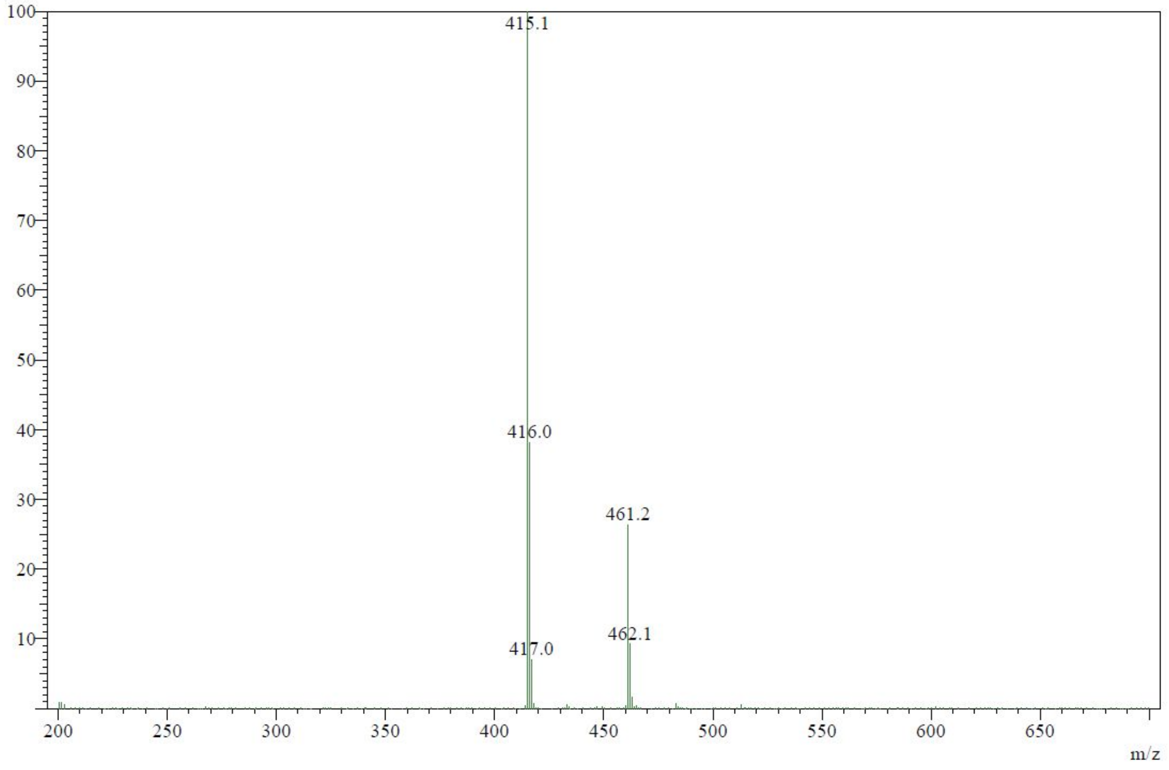

Figure S23. LC-MS spectra of compound **5**

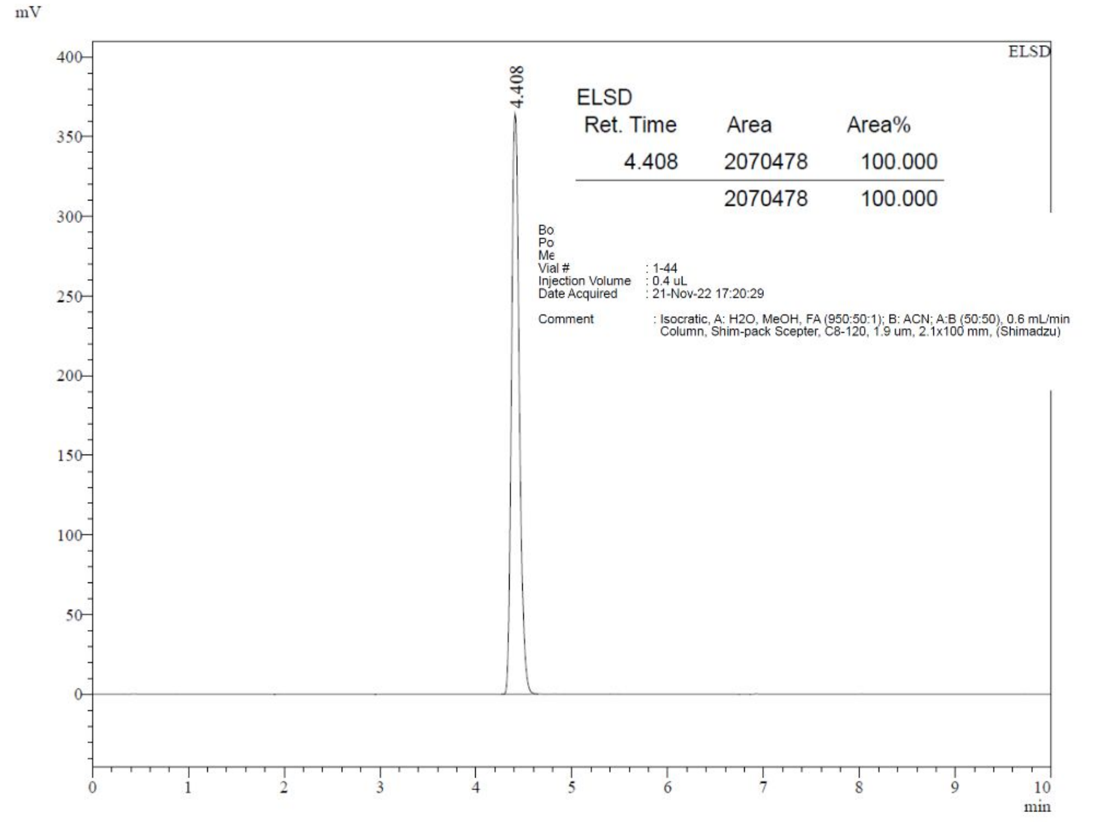

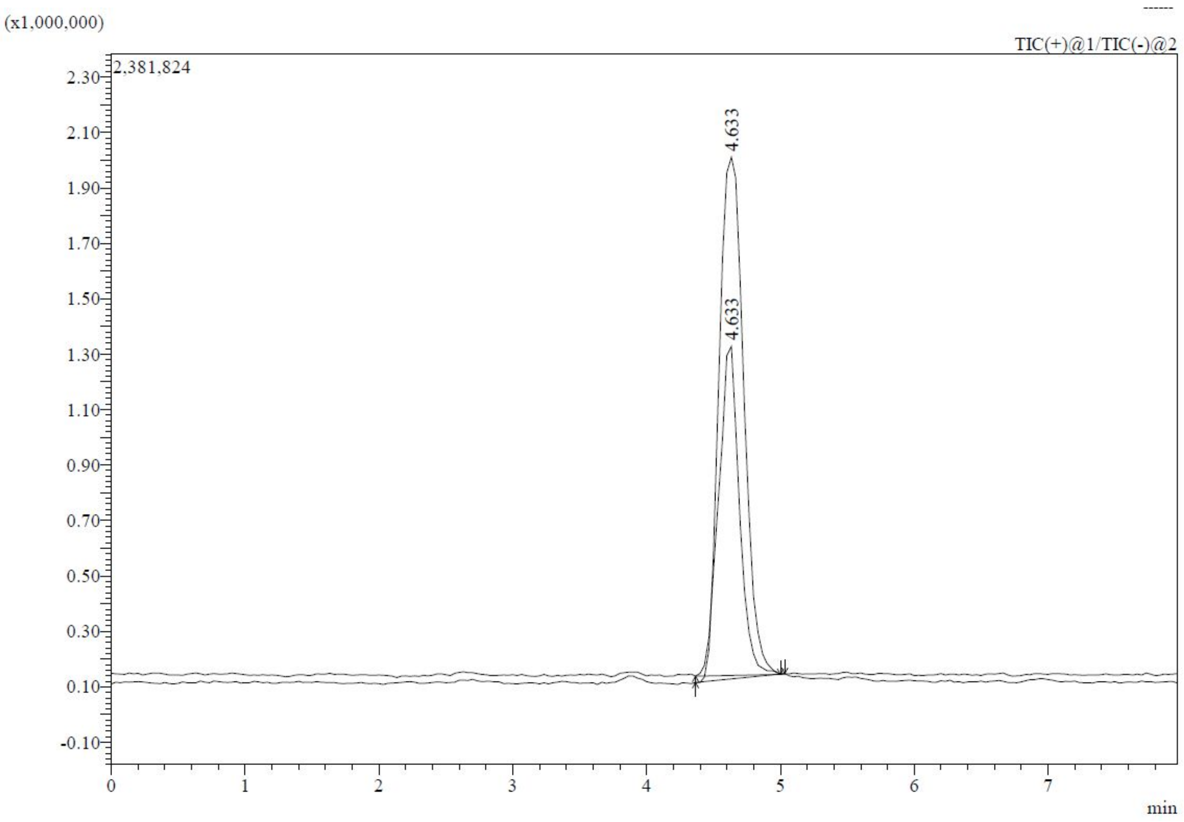

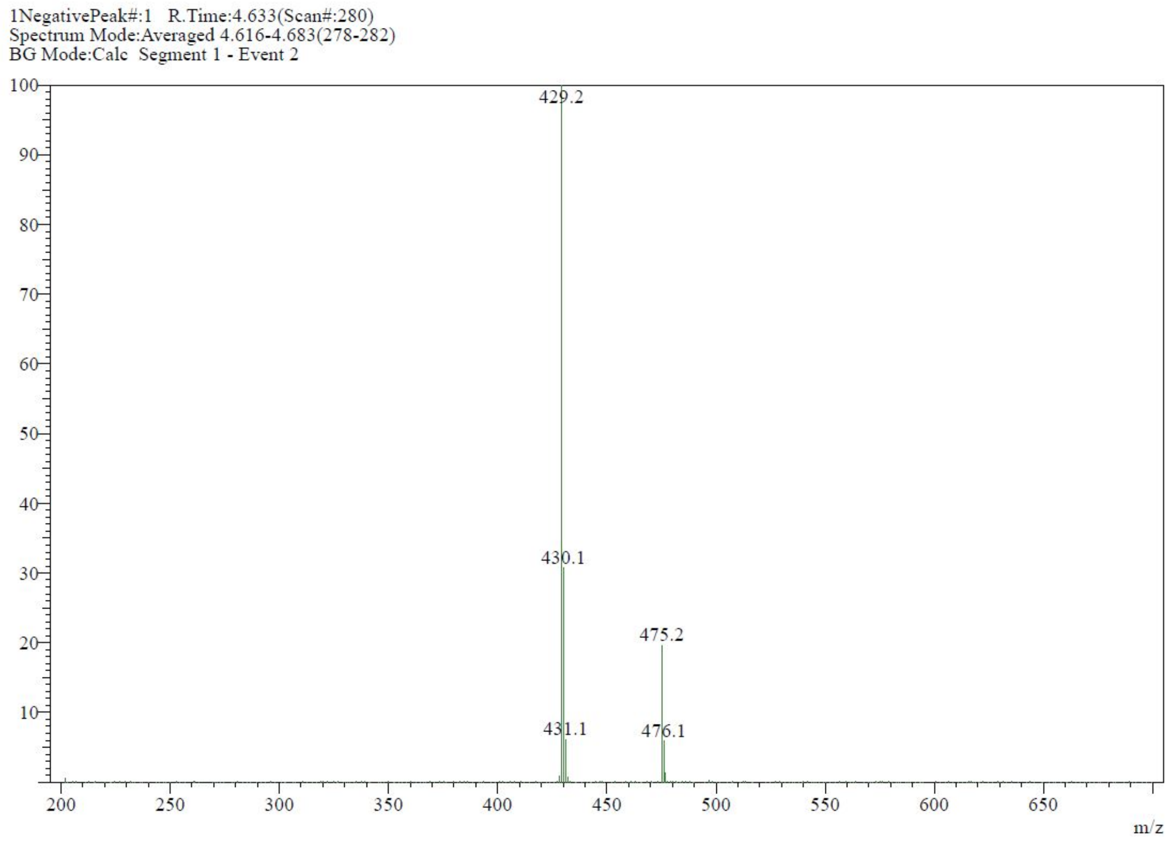

Figure S24. LC-MS spectra of compound **6**

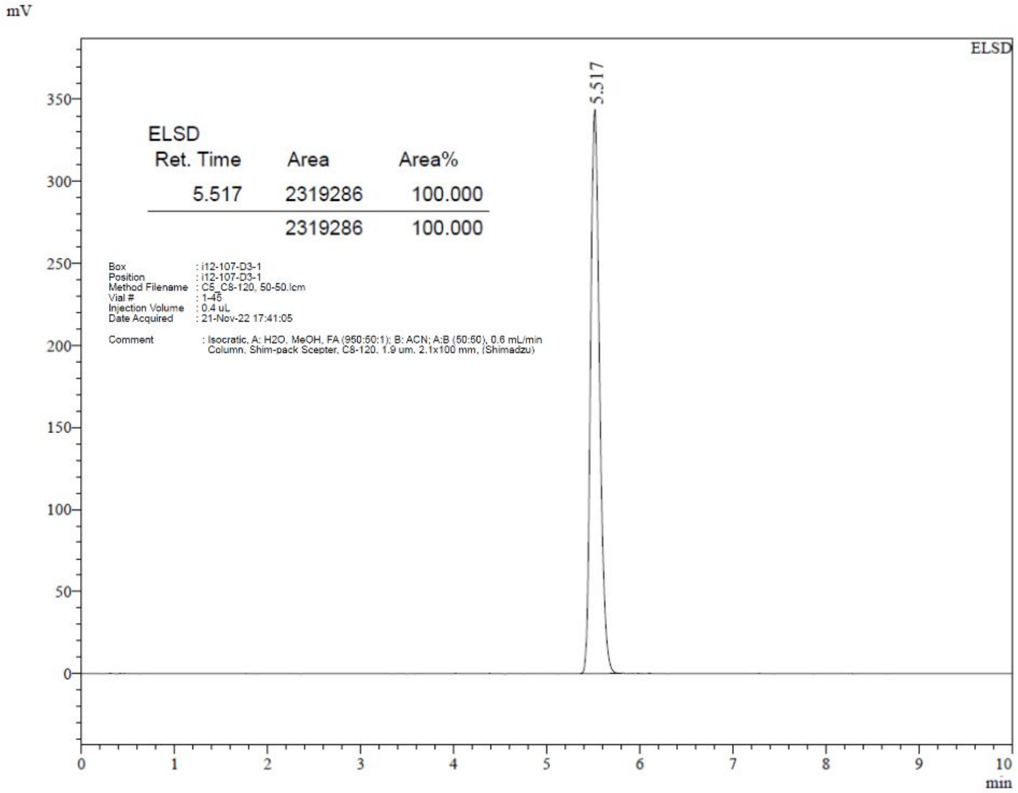

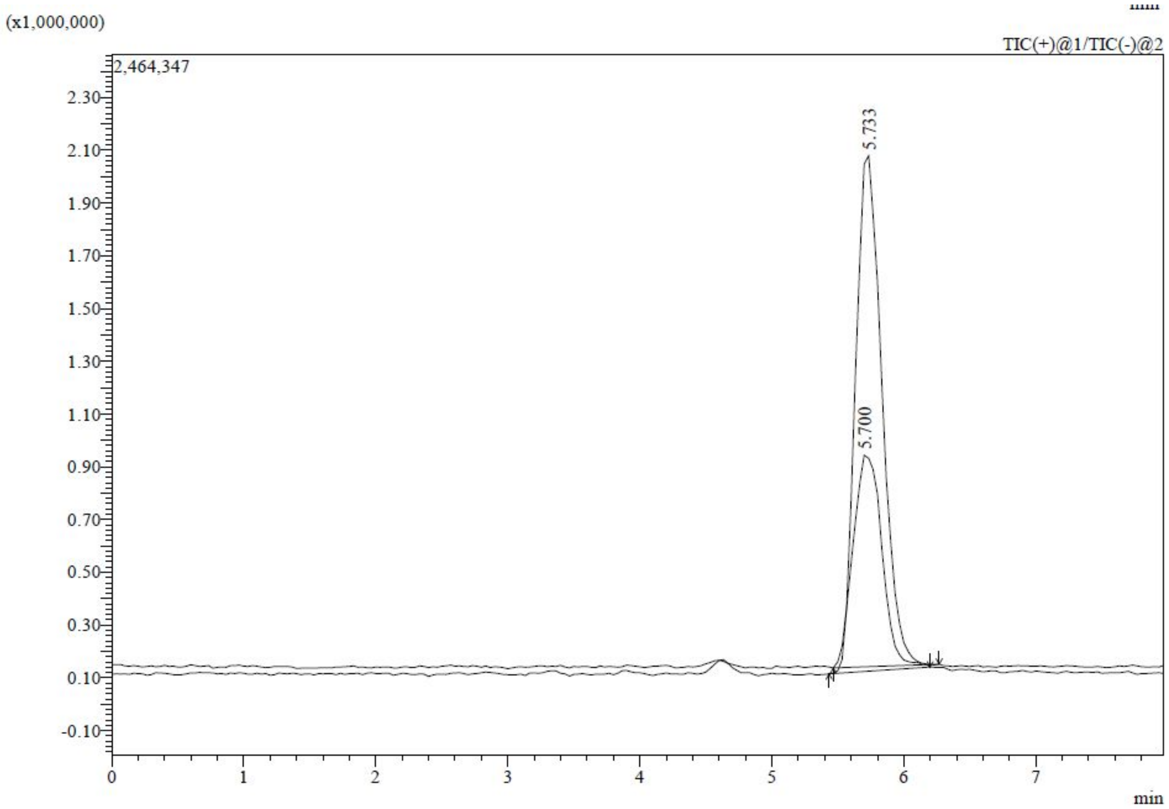

NegativePeak#:1 R.Time:5.700(Scan#:344)
Spectrum Mode:Averaged 5.683-5.750(342-346)
BG Mode:Calc Segment 1 - Event 2

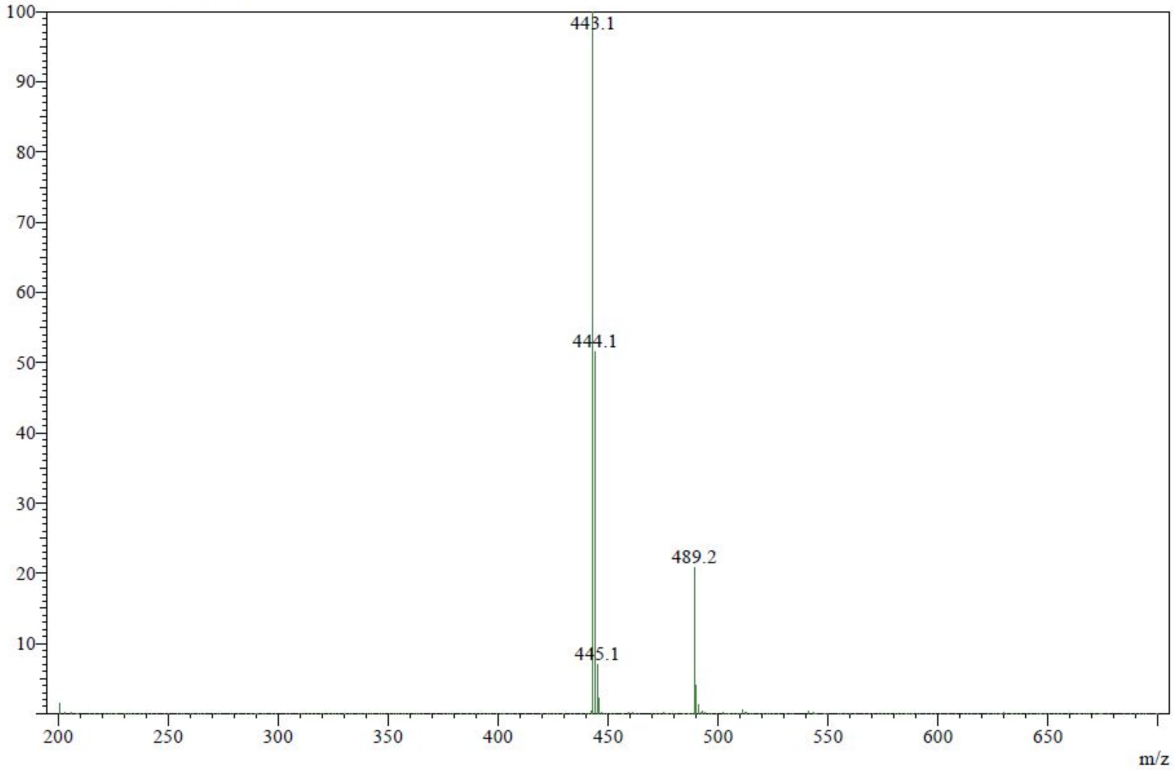

2 *In vitro* results

Table S1. *In vitro* inhibitory ability of the steroidal standards. Inhibition of 17β-HSD10 activity in the presence of the steroids at concentrations of 10 μM and 1 μM using 17β-estradiol as substrate. Values represents means ± SD (n = 4).

Steroid	Inhibition at 10 μM (%) (this study)	Inhibition at 1 μM (%) (this study)	Inhibition at 1 μM (%) Ayan et al. 2012
pregnenolone	5.9 ± 0.8	0.0 ± 3.1	11 ± 2.6
testosterone	15.7 ± 3.8	10.3 ± 5.1	0.0 ± 3.2
dihydrotestosterone	6.3 ± 2.0	0.0 ± 2.2	15 ± 0.8
androsterone	0.0 ± 2.4	3.2 ± 2.2	7.0 ± 5.9
epi-androsterone	7.0 ± 2.5	2.0 ± 4.9	6.0 ± 1.0
dehydroepiandrosterone	6.6 ± 3.5	5.4 ± 3.7	0.0 ± 3.2

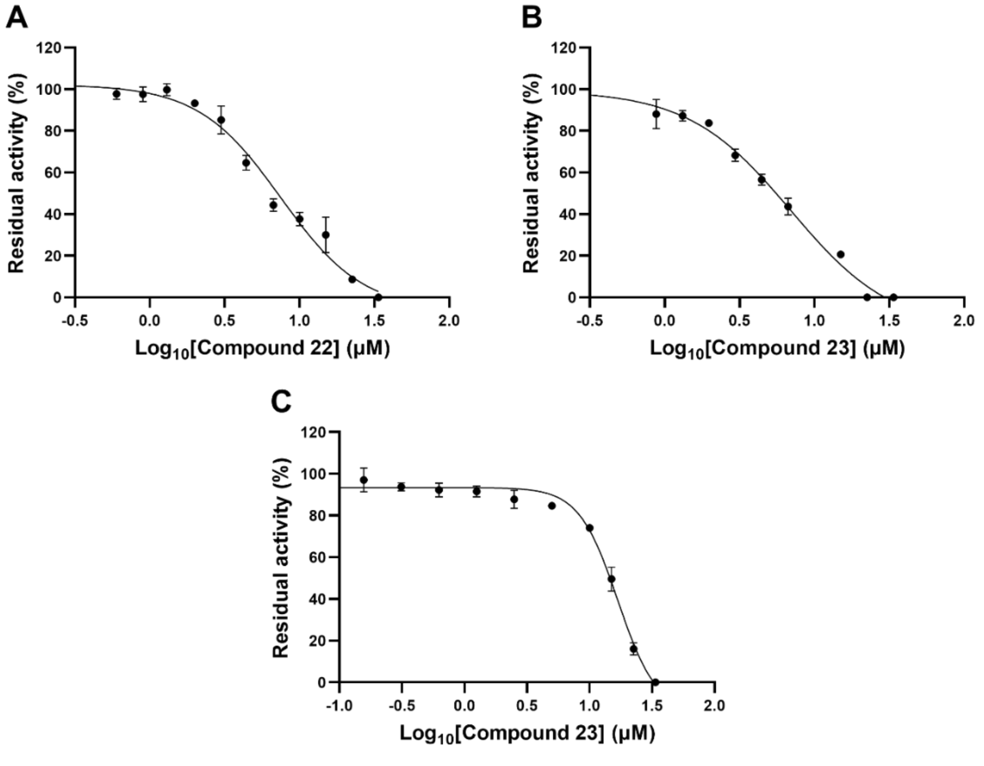

Figure S25. Dose-response curve for compound **23** (A and C) and compound **22** (B). The IC_{50} value with 17β-estradiol as substrate was determined 6.95 ± 0.35 μM for compound **22** (A) and 5.59 ± 0.25 μM for compound 23 (B). The IC_{50} value for compound **23** with allopregnanolone as substrate was determined 15.25 ± 0.39 μM (C). Presented data are shown as mean ± SEM (n = 4).
